# Loss of Renal Tubular PGC-1α Exacerbates Diet-Induced Renal Steatosis and Age-Related Urinary Sodium Excretion in Mice

**DOI:** 10.1371/journal.pone.0158716

**Published:** 2016-07-27

**Authors:** Kristoffer Svensson, Svenia Schnyder, Bettina Cardel, Christoph Handschin

**Affiliations:** Biozentrum, University of Basel, Klingelbergstrasse 50/70, CH-4056, Basel, Switzerland; Anatomy, SWITZERLAND

## Abstract

The kidney has a high energy demand and is dependent on oxidative metabolism for ATP production. Accordingly, the kidney is rich in mitochondria, and mitochondrial dysfunction is a common denominator for several renal diseases. While the mitochondrial master regulator peroxisome proliferator-activated receptor γ coactivator 1α (PGC-1α) is highly expressed in kidney, its role in renal physiology is so far unclear. Here we show that PGC-1α is a transcriptional regulator of mitochondrial metabolic pathways in the kidney. Moreover, we demonstrate that mice with an inducible nephron-specific inactivation of PGC-1α in the kidney display elevated urinary sodium excretion, exacerbated renal steatosis during metabolic stress but normal blood pressure regulation. Overall, PGC-1α seems largely dispensable for basal renal physiology. However, the role of PGC-1α in renal mitochondrial biogenesis indicates that activation of PGC-1α in the context of renal disorders could be a valid therapeutic strategy to ameliorate renal mitochondrial dysfunction.

## Introduction

The kidney is an important organ for the clearance of metabolic waste products from the blood, for maintaining body salt and fluid balance and for blood pressure homeostasis. This is achieved through passive filtration of plasma in the glomerulus, which is coupled to a system of transporters along the nephron, responsible for maintaining systemic nutrient- and salt homeostasis [1]. Tubular reabsorption is an energy-demanding process and the majority of ATP (~95%) in the kidney is produced through oxidative metabolism [2,3]. Consequently, mitochondrial density is highest in tubule segments associated with high basal transcellular transport rates, such as the proximal tubules and the thick loop of Henle [3]. The integral role of mitochondrial metabolism in renal function is underscored by the prevalence of renal dysfunction in patients suffering from mitochondrial cytopathies [2,4,5]. The peroxisome proliferator-activated receptor γ coactivator 1α (PGC-1α) is an important regulator of mitochondrial function [6]. While PGC-1α is highly expressed in the kidneys [7], the role of PGC-1α in renal physiology is so far unclear. To address this gap in knowledge, we have generated and characterized mice with a nephron-specific inducible PGC-1α knockout (NiPKO). Inactivation of PGC-1α in the kidney resulted in reduced expression of mitochondrial enzymes. NiPKO mice displayed a mild sodium-losing phenotype, but otherwise showed normal regulation of salt and water balance and blood pressure. Importantly, we found that PGC-1α is necessary for the transcriptional induction of lipid metabolic processes in the kidney upon high fat diet feeding. Consequently, NiPKO mice develop renal hypertriglyceridemia in this dietary context. Collectively, our results indicate a minor role for PGC-1α in basal renal physiology, mainly affecting age-related sodium excretion. Moreover, we observed a central role for PGC-1α in the transcriptional regulation of mitochondrial and metabolic processes in the kidney, most notably during high-fat diet feeding, with consequences on lipid accumulation and inflammation.

## Materials and Methods

### Animals and diets

Animals were housed in a conventional facility with a 12-h light/12-h dark cycle with free access to food and water. Mice were sacrificed by CO_2_ inhalation or terminal bleeding of anaesthetized animals. All experiments were performed in accordance with federal guidelines and were approved by the Kantonales Veterinäramt of Kanton Basel-Stadt under the consideration of 3R and to ensure minimal pain and stress in the animals. To generate nephron-specific inducible PGC-1α knockout (NiPKO) mice, we crossed mice with transgenic expression of the reverse tetracycline-dependent transactivator (rtTA) under control of the Pax8-promoter (Pax8-rtTA, a kind gift from Dr. Robert Koesters) [8] with transgenic (tetO-cre)-LC1 mice (obtained from the European Mouse Mutant Archive) [9]. These double-transgenic mice were subsequently crossed with mice having two floxed PGC-1α alleles (PGC-1αfl/fl, from internal breeding) [10]. While the PGC-1αfl/fl mice are in a C57BL/6 strain background, the LC1 and the Pax8-rtTA were in a mixed background. To account for that, littermate controls were used in all experiments. All experiments were performed in male mice. To induce the knockout of PGC-1α, doxycycline (DOX) (Sigma) (0.2 mg/mL) was administered *ad libitum* to the drinking water of 12 week old mice, with the addition of 2% sucrose (Sigma) to enhance palatability. After two weeks, mice were switched back to regular drinking water and were allowed at least one week of rest before experiments started. Recombination PCR was performed using primers binding to a region surrounding exons 3–5 of PGC-1α; forward 5’-TCCAGTAGGCAGAGATTTATGAC-3’, reverse 5’- CCAACTGTCTATAATTCCAGTTC-3’. This primer pair yields a product when exons 3–5 of PGC-1α are excised [10]. A control PCR reaction was performed using the following primers; forward 5’- ACCTGTCTTTGCCTATGATTC-3’, reverse 5’-CCAGTTTCTTCATTGGTGTG-3’. The experimental diets used for this study were either obtained from Harlan Teklad (low sodium diet (LSD, <0.02% Na) (TD.90228)) or from Research Diets Inc (high fat diet (HFD, 60 kcal% fat, D12492)). Blood pressure was measured in restrained conscious mice using a non-invasive tail-cuff blood pressure analyzer (BP-2000, Visitech system, Bioseb). The mice were acclimatized to this method for 5 consecutive days, measurements were taken for 5 days and values averaged similar to previous studies [11]. Body composition was measured using an EchoMRI-100™ analyzer (EchoMRI Medical Systems).

### Blood and urine analysis

For urine collection, mice were housed in single mouse metabolic cages (3600M021, Tecniplast) overnight (16 hours) or for 24 hours. Amount of food and water consumed was recorded, and urine was collected for further analysis. For plasma analysis, whole tail-vein blood was collected in Microvette tubes (Sarstedt). Levels of sodium, chloride, potassium, calcium, protein, urea and creatinine (enzymatic method) levels in urine and plasma were determined using an automated biochemical analyzer (Cobas c111 analyzer; Roche).

### RNA extraction and RT-PCR

Frozen tissue was homogenized using TRIzol reagent (Invitrogen) before addition of chlorophorm an centrifugation at 12’000 g for 15 min. Clear supernatant was removed and incubated with isopropanol before centrifugation at 12’000 g for 10 min. Then, pellet was washed twice with 75% ethanol and final pellet dissolved in water. RNA concentration was adjusted and cDNA synthesis was performed using 1 μg of total RNA. Semi-quantitative real-time PCR analysis was performed using Fast SYBR Green master mix (Applied Biosystems) on a StepOnePlus Real-Time PCR System (Applied Biosystems). Relative expression levels for each gene of interest were calculated with the ΔΔCt method, normalizing against mRNA levels of eukaryotic elongation factor 2 (*eEF2*) or TATA-binding protein (*Tbp*). A list of primer sequences used in this study can be found in S1 Table.

### Gene expression array

Gene expression analysis of control and NiPKO kidneys was performed using 4 samples per group, with the Affymetrix GeneChip Mouse Gene 2.0 ST microarray. Transcripts were considered significantly altered if having a p-values <0.05 and a fold-change of >1.2. Pathway enrichment analysis was performed using Babelomics [12] and the Kyoto Encyclopedia of Genes and Genomes (KEGG) database [13]. KEGG terms were considered significantly enriched with a cut-off of p-values<0.05. Integrated System for Motif Activity Response Analysis (ISMARA) [14] was used to predict the core transcription factors driving the transcriptional changes observed in our gene expression array.

### Mitochondrial DNA (mtDNA) analysis

Analysis of mtDNA was performed similar to previous descriptions [15]. Frozen tissue was homogenized and DNA was extracted via a standard phenol/chloroform extraction protocol. DNA concentration was adjusted and the relative amount of mtDNA (primers against mtDNA D-loop region) to nuclear DNA (*Ndufv1* primers) was analyzed using Fast SYBR Green master mix (Applied Biosystems) on a StepOnePlus Real-Time PCR System (Applied Biosystems).

### Protein isolation and immunoblotting

Frozen tissues were homogenized in radioimmunoprecipitation assay (RIPA) buffer and after centrifugation at 13’000 g protein concentration of the supernatant was measured using the Bradford method (Biorad). Equal amounts of proteins were separated on SDS-PAGE under reducing conditions and transferred to a nitrocellulose membrane (Whatman). Proteins of interest were detected using the following primary antibodies: Mitoprofile (MS604, MitoSciences), NKCC2 (sc-133823, Santa Cruz Biotechnology), NCCT (sc-21554, Santa Cruz Biotechnology) and eEF2 (2332; Cell signaling). Densitometric analysis of immunoblots was performed on 6 individual samples using Image-J software and a representative selection from this group is presented in each figure.

### Histology

After extraction, kidneys were fixed in 4% paraformaldehyde/PBS at 4°C overnight. Kidneys were subsequently dehydrated, embedded in paraffin and 5 μm thick sections were cut using a microtome. For general histology, a Periodic Acid-Schiff (PAS) staining was performed according to the manufacturer’s instructions (PAS Kit, Sigma).

### Isolation of triglycerides from kidney

Triglycerides were isolated from snap-frozen kidney as previously described (Svensson et al., 2015). Briefly, frozen kidney tissue was homogenized in a 2:1 chloroform/methanol mixture. The organic phase was subsequently dried under N2, re-suspended and cleaned on a solid phase extraction column. Triglycerides were measured using a commercial enzymatic kit (TG PAP 150, BioMérieux) and normalized to the initial weight of the tissue used for extraction.

### Statistical analysis

All data are presented as means ± SEM. Unpaired student two-tailed t-test was used to determine differences between groups.

## Results

### Knockout of PGC-1α in renal tubular cells results in a mild salt-losing phenotype

**Fig 1:**
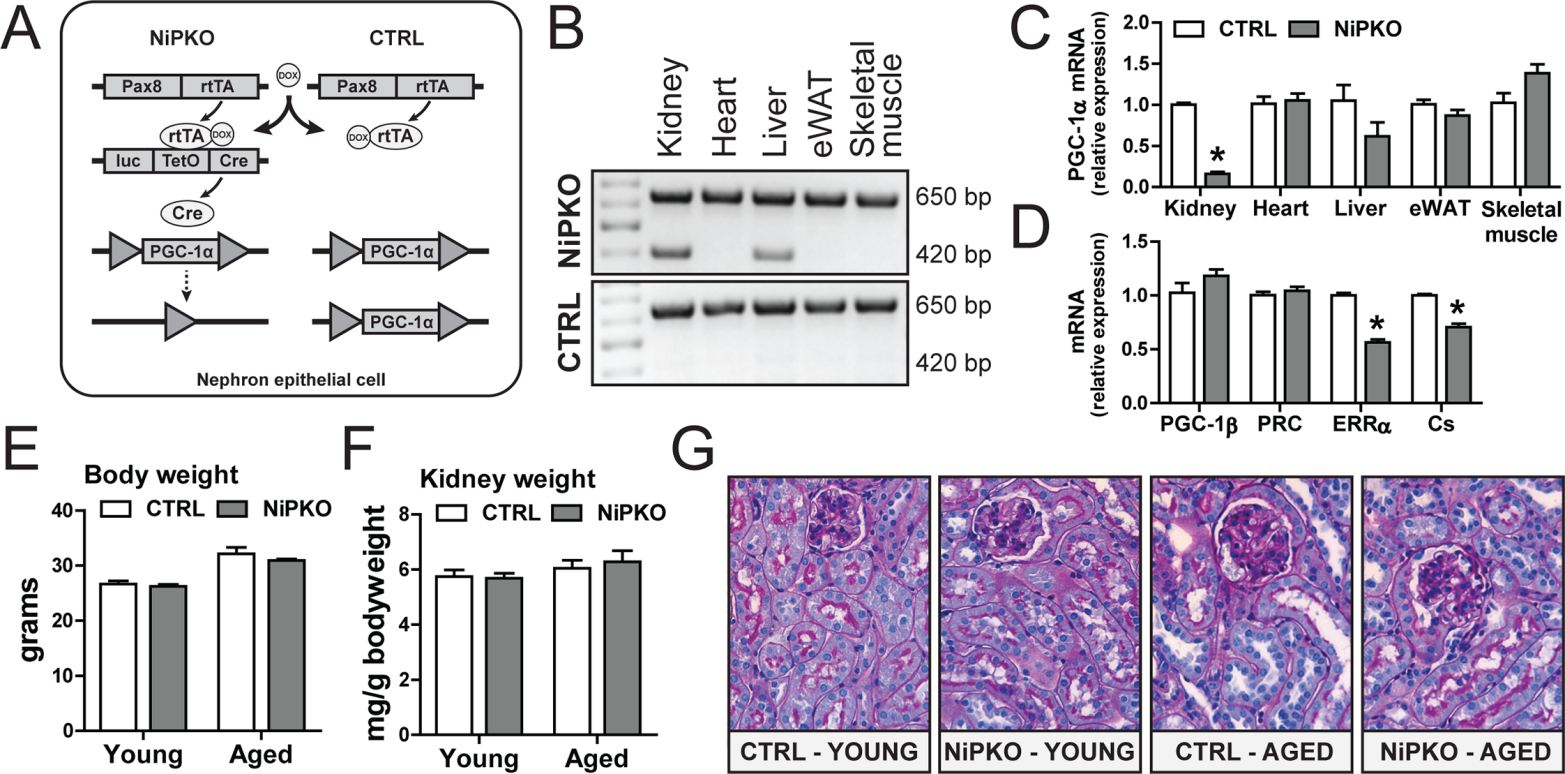
Kidney-specific inactivation of PGC-1α in NiPKO mice. (A) Nephron-specific inducible PGC-1α knockout (NiPKO) mice were generated by crossing transgenic Pax8rtTA-(tetO-cre)-LC1 mice with mice having two floxed PGC-1α alleles (PGC-1αfl/fl). Knockout was induced through doxycycline administration for 14 days. (B) Representative recombination PCR for PGC- 1α on DNA extracted from kidney, heart, liver, epididymal white adipose tissue (eWAT) and skeletal muscle. The amplified products for the wild-type and knockout alleles are approximately 650 bp and 420 bp, respectively. (C) mRNA levels of PGC-1α in kidney, heart, liver, eWAT and skeletal muscle, normalized to eukaryotic elongation factor 2 (eEF2) mRNA levels (n = 5–7). (D) mRNA levels of indicated genes in kidney normalized to eEF2 mRNA levels (n = 7–8). (E) Body weight and (F) average kidney weight normalized to body weight of mice at 1 and 12 months after doxycycline administration (n = 6–16). (G) Representative pictures of kidney histology at 1 and 12 months after doxycycline administration (n = 3). Error bars represent mean ±SEM. Significant differences (p-value <0.05) between genotypes are indicated by an asterisk (*).

**Fig 2:**
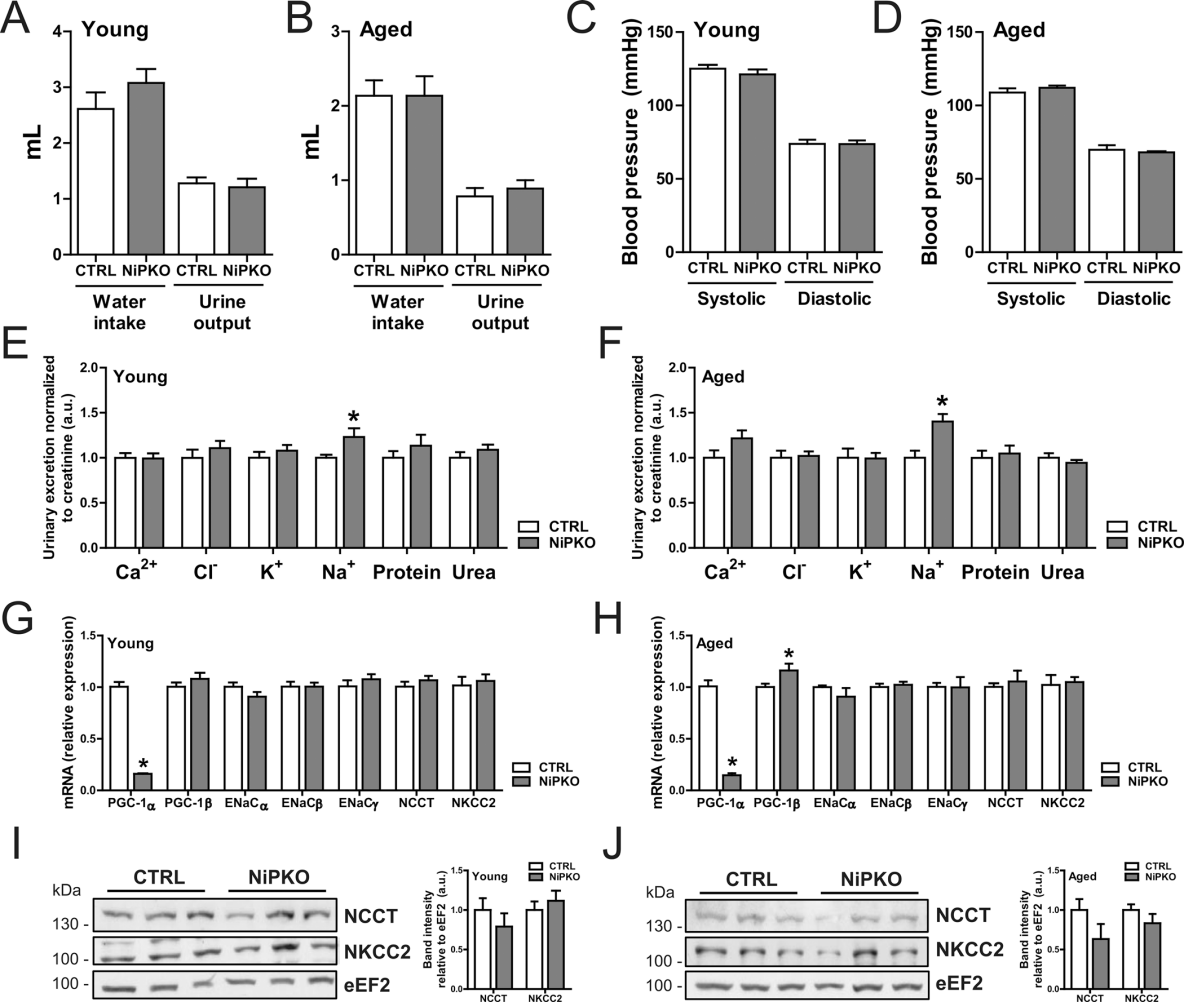
NiPKO mice display elevated sodium-excretion in urine. (A-B) Water intake and urine output in mice over 24 hours, at (A) 1 month and (B) 12 months after doxycycline administration (n = 8–13). (C-D) Systolic and diastolic blood pressure measured via tail-cuff photoplethysmography at (C) 1 month and (D) 12 months after doxycycline administration (n = 6–8). (E-F) Urinary levels of calcium (Ca2+), chloride (Cl-), potassium (K+), sodium (Na+), protein and urea over 24 hours, normalized to urinary excretion of creatinine, at (E) 1 month and (F) 12 months after doxycycline administration (n = 8–13). (G-H) mRNA levels of indicated genes in kidney normalized to *Tbp* mRNA levels, at (G) 1 month and (H) 12 months after doxycycline administration (n = 6). (I-J) Representative immunoblots of NCCT, NKCC2 and eEF2 in kidney. Bar graph shows quantification of band intensities of NCCT and NKCC2 relative to eEF2 (n = 6). Error bars represent mean ±SEM. Significant differences (p-value<0.05) between genotypes are indicated by an asterisk (*).

To inactivate PGC-1α in the kidney, we generated nephron-specific inducible PGC-1α knockout (NiPKO) mice by crossing transgenic Pax8rtTA-(tetO-cre)-LC1 animals [8,9] with mice harboring floxed PGC-1α alleles (PGC-1αfl/fl) [10] (Fig 1A). Through administration of doxycycline (DOX), this system allows specific inactivation of PGC-1α in the complete renal tubule system while sparing the glomerulus [8]. Successful recombination of exon 3–5 of PGC-1α was confirmed in kidney from NiPKO mice after DOX-administration (Fig 1B). In line with a low *Pax8* expression in liver [8], we also detected recombination of PGC-1α in liver of NiPKO mice (Fig 1B). However, while PGC-1α mRNA expression was strongly diminished in the kidney, there was no significant reduction in PGC-1α transcript levels in the liver (Fig 1C). All other tissues tested showed unchanged PGC-1α mRNA levels between control and NiPKO mice (Fig 1C). The inactivation of PGC-1α in kidney did not affect expression of the related family members PGC-1β and PGC1-related coactivator (PRC) (Fig 1D), but resulted in significantly reduced transcript levels of known PGC-1α target genes such as estrogen related receptor α (ERRα) and citrate synthase (Cs) (Fig 1D). Next, the phenotype of the NiPKO mice was investigated in young mice (1 month after DOX administration) and aged mice (12 months after DOX administration). Inactivation of PGC-1α in kidney led to no significant changes in either body weight (Fig 1E) or relative kidney weight (Fig 1F) in either age category. Moreover, kidneys from NiPKO mice displayed normal histological features compared to control mice (Fig 1G, S1A and S1B Fig). Subsequently, we analyzed salt and water homeostasis and blood pressure in control and NiPKO mice. Inactivation of PGC- 1α in the kidney did not affect water intake or urine output in either young (Fig 2A) or aged (Fig 2B) NiPKO mice compared to control mice. Similarly, systolic and diastolic blood pressure was indistinguishable between the groups at these time points (Fig 2C and 2D). Interestingly, NiPKO mice displayed a significant increase in urinary sodium excretion when normalized to urinary creatinine excretion in both young (Fig 2E) and aged animals (Fig 2F). However, there was no significant difference for the urinary excretion of chloride, potassium and calcium or for proteins and urea (Fig 2E and 2F), or in plasma levels of sodium or any of the above-mentioned parameters (S2A and S2B Fig). The increased urinary sodium excretion was not connected to alterations in transcript levels of the epithelial Na+ channel α (ENaCα), ENaCβ or ENaCγ subunits in either young or aged mice (Fig 2G and 2H). Likewise, no differences in transcript (Fig 2G and 2H) or protein (Fig 2I and 2J) levels of the Na+-Cl− cotransporter (NCCT) or Na+-K+-Cl− cotransporter (NKCC2) were detected. These data indicate that NiPKO mice display a largely normal regulation of water intake, urine output and blood pressure, but display a mild sodium-losing phenotype in the basal state.

### Aged NiPKO mice cannot adapt their sodium excretion during low salt diet feeding

**Fig 3:**
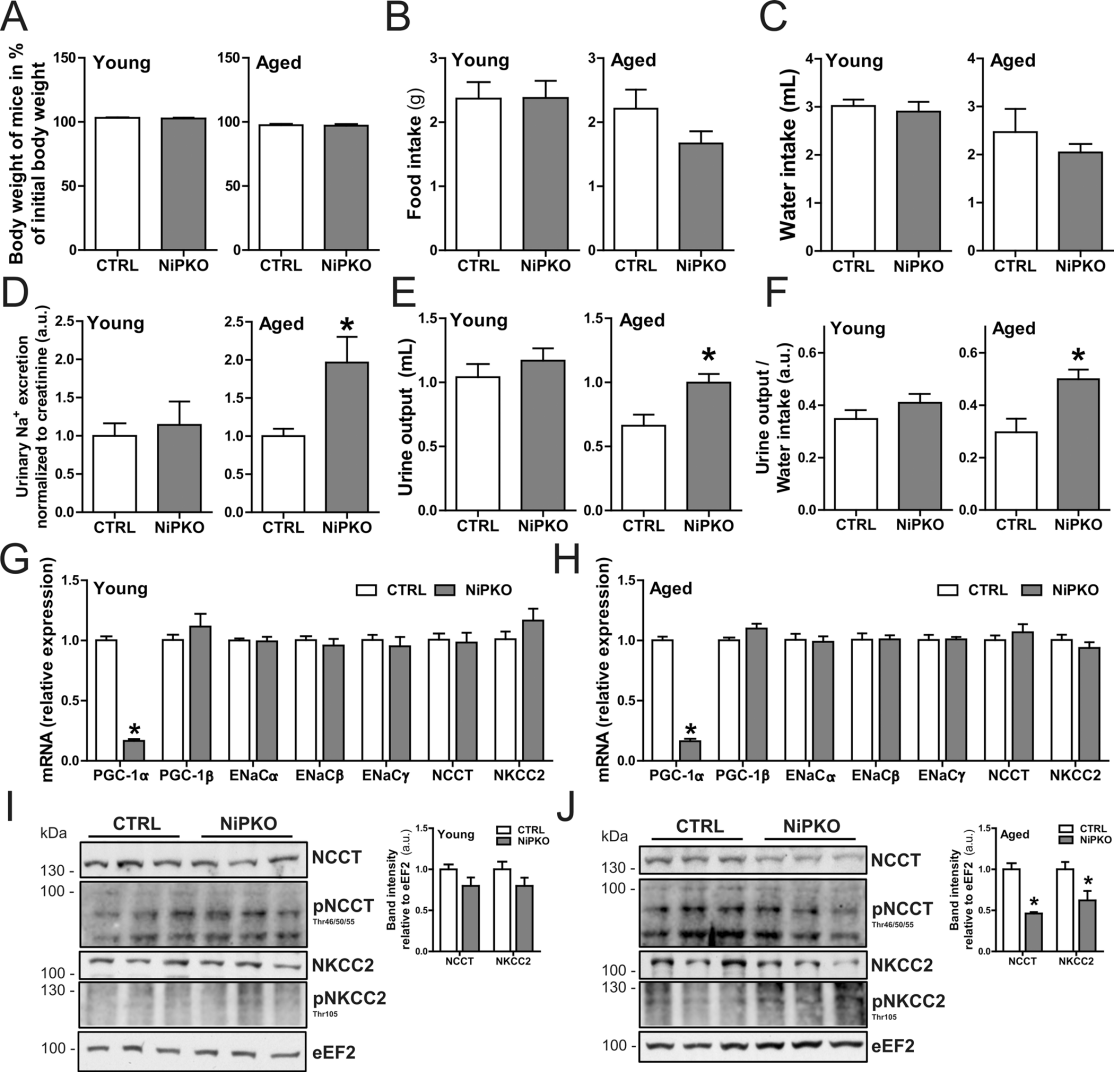
Aged NiPKO mice cannot adapt their salt- and water homeostasis to a reduced dietary salt intake. Control (CTRL) and NiPKO mice were fed a standardized diet containing <0.02% NaCl (low salt diet, LSD) for 5 weeks, starting at either 1 month or 12 months after doxycycline administration. (A) Body weight at end of LSD feeding period expressed as percentage of initial body weight (n = 6–8). (B) Food intake over 24 hours (n = 6–8). (C) Water intake over 24 hours (n = 6–8). (D) Urinary sodium (Na+) excretion normalized to urinary creatinine excretion (n = 6–8). (E) Urine output over 24 hours (n = 6–8). (F) Urine-to-water ratio over 24 hours (n = 6–8). (G-H) mRNA levels of indicated genes in kidney normalized to *Tbp* mRNA levels, at (G) 1 month and (H) 12 months after DOX administration (n = 6). (I-J) Representative immunoblots of NCCT, p-NCCT (Thr45/50/55), NKCC2, p-NKCC2 (Thr105) and eEF2 in kidney. Bar graph shows quantification of band intensities of total NCCT and NKCC2 relative to eEF2 (n = 6). Error bars represent mean ±SEM. Significant differences (p-value <0.05) between genotypes are indicated by an asterisk (*).

The small elevation in urinary sodium excretion in NiPKO mice indicates a role for PGC-1α in the maintenance of renal salt homeostasis. To test this notion, we exposed control and NiPKO animals to a dietary salt stress and assessed salt- and water homeostasis. Control and NiPKO mice were administered a standardized diet containing <0.02% NaCl (low salt diet, LSD) for 5 consecutive weeks, starting at either 1 or 12 months after DOX-administration. LSD-feeding did not affect body weight in either group (Fig 3A) and there was no significant difference in food intake (Fig 3B) or water intake (Fig 3C) between LSD-fed control and NiPKO mice. Interestingly, despite the mild salt-losing phenotype in the basal state (Fig 2E), young NiPKO mice could adapt their urinary sodium excretion to the same extent as control mice during LSD feeding (Fig 3D). Consequently, there were no differences in either urine output (Fig 3E) or urine-to-water ratio (Fig 3F) between young control and NiPKO mice with LSD feeding. In contrast to the young mice however, aged NiPKO mice displayed elevated urinary sodium levels (Fig 3D) with a concomitant elevated urine output (Fig 3E) and urine-to-water ratio (Fig 3F) in aged NiPKO mice compared to control mice with LSD feeding. Collectively, these data indicate that while young NiPKO mice can adapt their reabsorption of urinary sodium during a reduced dietary salt intake to the same extent as control mice, this process is impaired in aged NiPKO mice. Similar to chow-fed mice (Fig 2G and 2H), there were no differences in transcript levels of the sodium transporters ENaC (α/β/γ subunits), NCCT or NKCC2 between young or aged control and NiPKO mice (Fig 3G and 3H). Interestingly, while protein levels of NCCT and NKCC2 were comparable between young LSD-fed control and NiPKO mice (Fig 3I), aged NiPKO mice displayed significantly reduced renal protein levels of both NCCT and NKCC2 compared to control mice while the phosphorylated forms of NCCT and NKCC2 were not different between the groups at either age (Fig 3I and 3J). Importantly, the reduced levels of the renal sodium-transporters NCCT and NKCC2 in aged NiPKO mice could explain the salt losing phenotype specifically in this group. However, since transcription of the genes encoding these channels was unaltered (Fig 3G and 3H), the reduced protein levels of NCCT and NKCC2 is most likely an indirect effect of PGC-1α inactivation in aged mice.

### PGC-1α regulates mitochondrial gene transcription in the kidney

**Fig 4:**
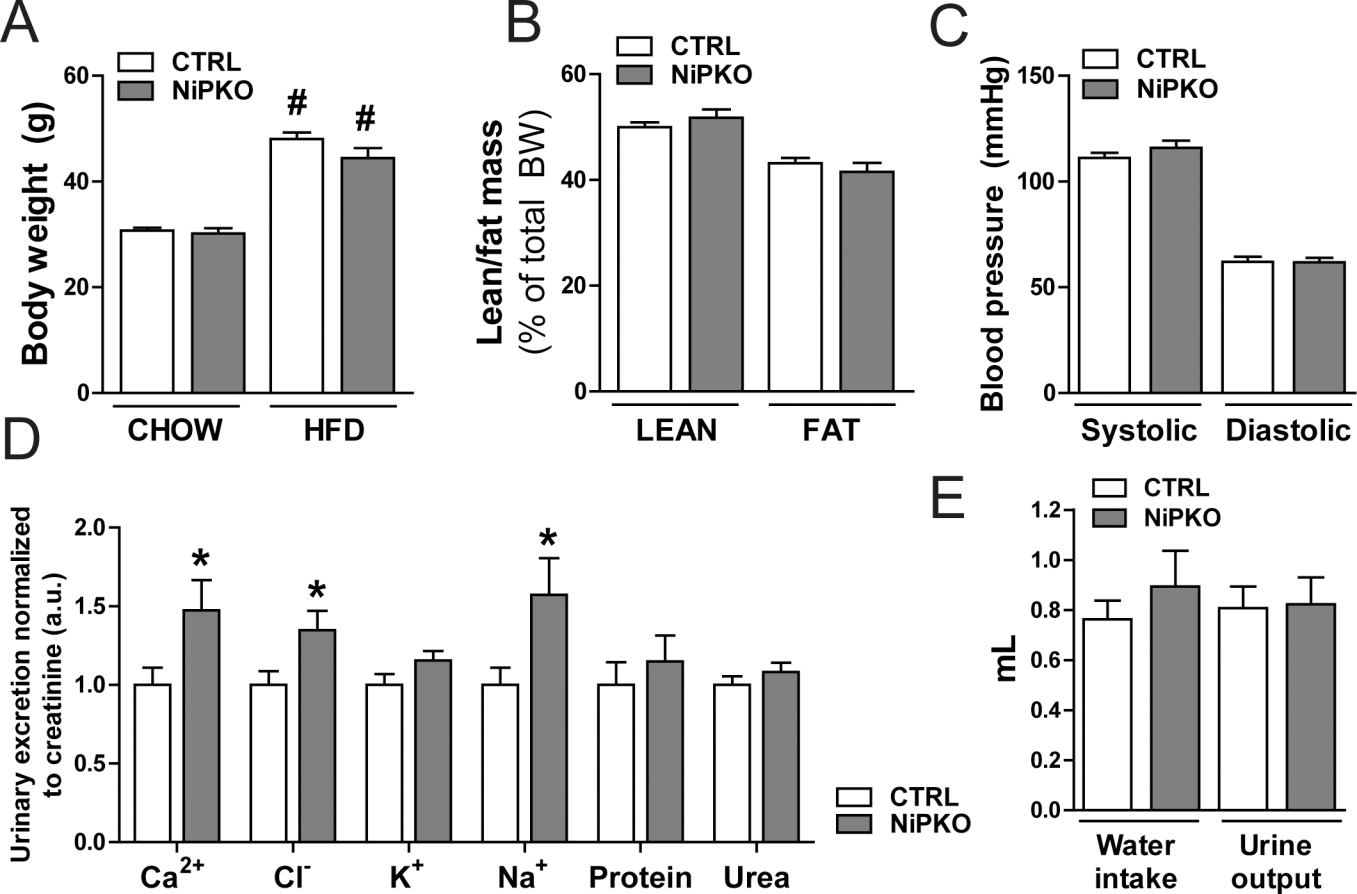
High fat diet-induced changes in control and NiPKO mice. Control (CTRL) and NiPKO mice were fed either a chow-diet (CHOW, 10 kcal% fat) or a high fat diet (HFD, 60 kcal% fat) for 5 months, starting at 2 weeks after DOX-administration. (A) Body weight at the end of chow or HFD feeding period (n = 10–15). (B) Body composition expressed as percentage lean and fat mass of total body weight of HFD-fed mice (n = 16). (C) Systolic and diastolic blood pressure measured via tail- cuff photoplethysmography of HFD-fed mice (n = 10). (D) Urinary levels of calcium (Ca2+), chloride (Cl-), potassium (K+), sodium (Na+), protein and urea over 16 hours, normalized to urinary excretion of creatinine, at the end of HFD feeding period (n = 9). (E) Water intake and urine output over 16 hours of HFD-fed mice (n = 15–16). Error bars represent mean ±SEM. Significant differences (p-value <0.05) between genotypes are indicated by an asterisk (*) and between chow- and HFD-fed groups by a number sign (#).

PGC-1α is an important transcriptional regulator of mitochondrial and metabolic processes [6]. Consequently, we were interested in how inactivation of PGC-1α affects the renal transcriptome in mice fed a chow diet and in the context of high fat diet (HFD)-induced metabolic stress. Accordingly, starting one week after the end of the DOX administration, control and NiPKO mice were fed either a chow diet or a HFD (60 kcal% fat) for five months. At the end of this period, HFD-fed control and NiPKO mice were significantly heavier than the chow fed cohorts (Fig 4A). HFD-fed control and NiPKO mice gained a similar amount of weight and displayed no difference in body composition (Fig 4A and 4B). Moreover, systolic and diastolic blood pressure was unaltered between genotypes during HFD-feeding (Fig 4C). Similar to chow fed mice (Fig 2E and 2F), we could confirm the salt-losing phenotype in HFD-fed NiPKO mice (Fig 4D). Interestingly, HFD- fed NiPKO mice displayed enhanced urinary excretion not only of sodium, but also of chloride and calcium (Fig 4D). Despite the elevated levels of electrolytes in the urine, HFD-fed NiPKO mice displayed unaltered water intake and urine output compared to control mice (Fig 4E). Moreover, differences in food intake cannot account for the modulation of chloride and calcium (S3 Fig). Overall, HFD-feeding exacerbated the loss of ions in the urine, but did not significantly impact the overall ability of NiPKO mice to adjust their salt and water homeostasis and blood pressure regulation. Accordingly, the transcriptional regulation of several transporters by genotype or diet remains relatively mild (S4 Fig).

**Fig 5:**
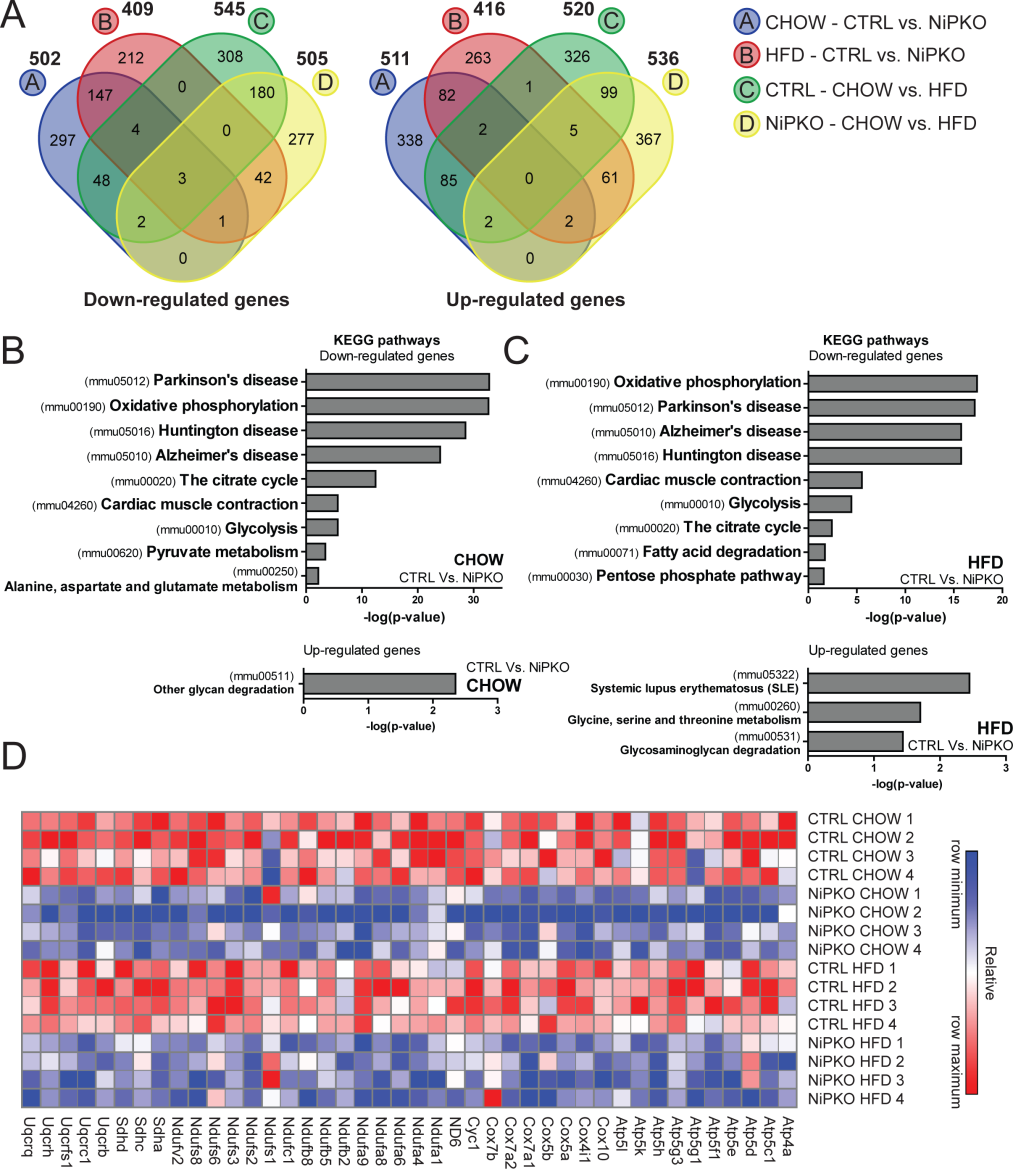
PGC-1α regulates transcription of mitochondrial and metabolic genes in kidney. (A) VENN diagrams displaying the number of unique or overlapping down- or up-regulated genes (p<0.05 and fold change >1.2 cut-off) in either chow- or HFD-fed control (CTRL) or NiPKO mice. (B-C) Top significant terms for KEGG pathway enrichment analysis of down- or up-regulated genes in kidneys from (B) chow- fed NiPKO mice compared to CTRL mice or in (C) HFD-fed NiPKO mice compared to CTRL mice. (D) Heat map generated using probe set intensities for transcripts associated with the KEGG-category “*Oxidative phosphorylation*” in Fig B and C, for CHOW and HFD-fed CTRL and NiPKO mice. Row minimum = -3, row maximum = 3 fold change.

**Fig 6:**
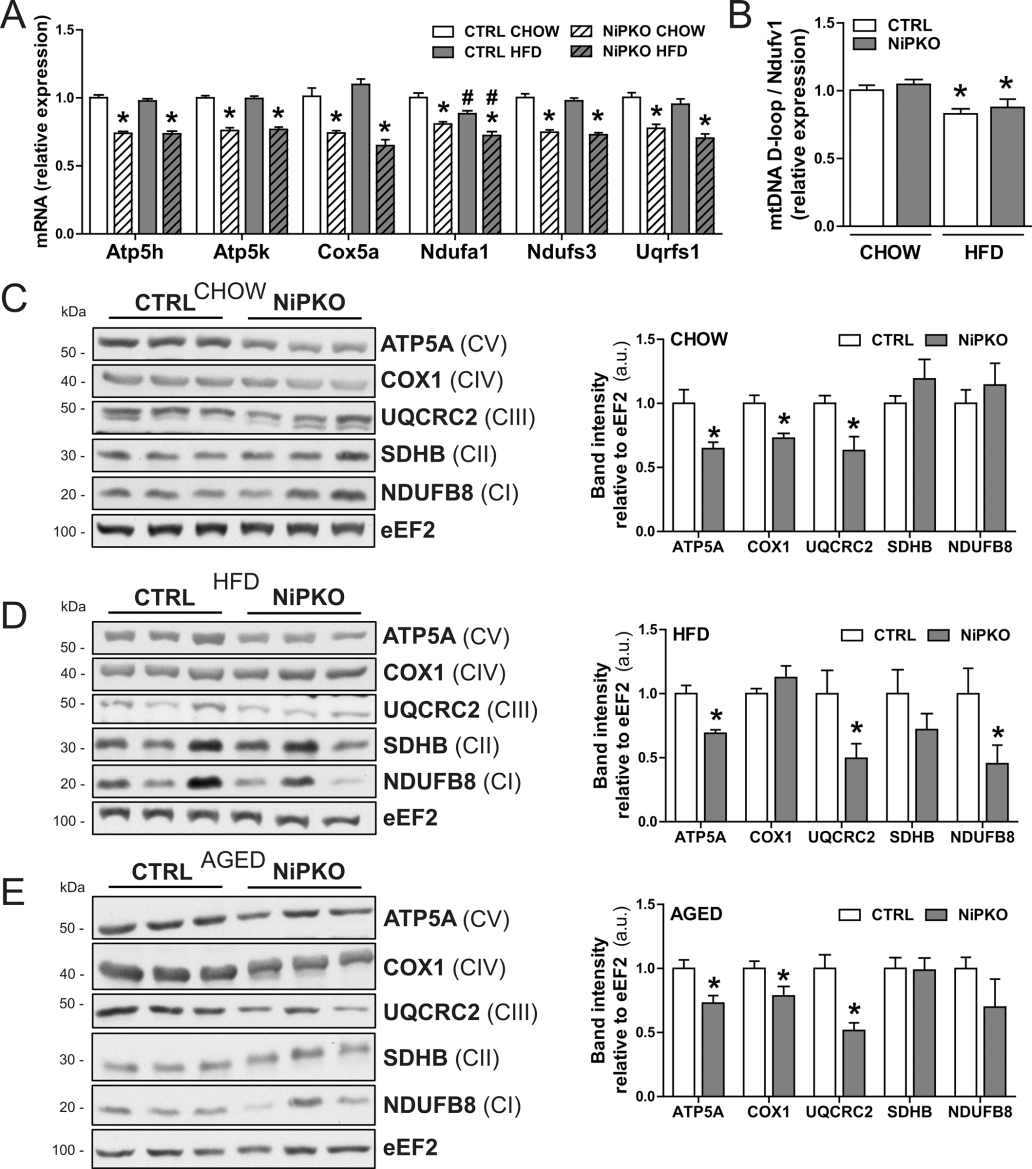
NiPKO mice display reduced levels of mitochondrial enzymes in kidney. (A) mRNA levels of indicated genes in kidney normalized to *eEF2* mRNA levels, in either CHOW- or HFD- fed control (CTRL) or NiPKO mice (n = 7–8). (B) Mitochondrial DNA (mtDNA) content expressed as the relative content of mtDNA D-loop compared to the nuclear *Ndufv1* gene (n = 7). (C-E) Representative immunoblots of ATP5A, COX1, UQCRC2, SDHB, NDUFB8 and eEF2 in kidney. Bar graph shows quantification of band intensities relative to eEF2 for either (C) chow-fed, (D) HFD-fed or (E) aged chow- fed CTRL and NiPKO mice (n = 6). CI, CII, CIII, CIV and CV represents subunits of electron transport chain complex I, II, III, IV and V, respectively. Error bars represent mean ±SEM. Significant differences (p-value <0.05) between genotypes are indicated by an asterisk (*) and between chow- and HFD-fed groups by a number sign (#).

Next, we focused on changes in the renal transcriptome by performing a gene expression array on kidney from chow- and HFD-fed control and NiPKO mice. A total of 1013 unique genes (502 down, 511 up) were significantly changed in kidneys from chow-fed NiPKO mice compared to chow-fed control mice. For HFD- fed NiPKO mice compared to HFD-fed control mice, 825 genes (409 down, 416 up) were significantly changed (Fig 5A, S2 and S3 Tables). In control mice, 1065 unique genes (545 down, 520 up) were significantly altered with HFD-feeding while in NiPKO mice, the same comparison yielded 1041 significantly changed genes (505 down, 536 up) (Fig 5A, S2 and S3 Tables). To elucidate the functional networks regulated by PGC-1α in kidney, we performed pathway enrichment analysis on differentially regulated transcripts in NiPKO compared to control mice, using the Kyoto Encyclopedia of Genes and Genomes (KEGG) database [13]. Terms associated with metabolic and mitochondrial processes, including *oxidative phosphorylation (mmu00190)*, *the citrate cycle (mmu00020)* and *glycolysis (mmu00010)* were significantly enriched amongst the downregulated genes in NiPKO mice both in the chow-fed and HFD-fed cohorts (Fig 5B and 5C, S4 Table). Additionally, the KEGG pathway enrichment analysis revealed terms such as *Parkinson’s disease (mmu05012)*, *Huntington disease (mmu05016)* and *Alzheimer’s disease (mmu05010)*, which contain many genes encoding mitochondrial proteins (e.g. cytochrome oxidase 7a1/*Cox7a1*, *ATP-synthase subunit 5h/Atp5h* and cytochrome c/*Cycs*) to be downregulated in NiPKOs (Fig 5B and 5C, S4 Table). Hence, inactivation of PGC-1α in kidney leads to a robust reduction in the transcription of genes associated with mitochondrial function and metabolism. In particular, in the KEGG-category *oxidative phosphorylation (mmu00190)*, many genes encoding proteins of the mitochondrial electron transport chain were down- regulated in both chow- and HFD-fed NiPKO mice compared to control mice (Fig 5D). We could furthermore validate the down-regulation of some selected mitochondrial oxidative phosphorylation components by RT-PCR (Fig 6A). Thus, in analogy to other tissues (Villena, 2015), PGC-1α is an important transcriptional regulator of mitochondrial genes also in the kidney. To investigate whether inactivation of PGC-1α affects mitochondrial biogenesis in the kidney, we then assessed mitochondrial DNA (mtDNA) content and mitochondrial protein expression in chow- and HFD-fed NiPKO and control mice, respectively. HFD-feeding reduced relative mtDNA content in the kidney, but there was no difference between genotypes (Fig 6B). On the other hand, there was a robust reduction in protein levels of ATP5A and UQCRC2 in the kidney of chow-fed (Fig 6C), HFD-fed (Fig 6D) and aged chow-fed (Fig 6E) NiPKO mice compared to control mice. In chow-fed and aged chow-fed mice, COX1 levels were also significantly reduced in NiPKO compared to control mice (Fig 6C and 6E), while NADH:ubiquinone oxidoreductase subunit B8 (NDUFB8) protein levels were specifically reduced in kidneys from HFD-fed NiPKO mice (Fig 6D). Other mitochondrial proteins tested were not significantly altered between control and NiPKO mice (Fig 6C–6E). Taken together, PGC-1α is important for the transcriptional regulation of metabolic and mitochondrial gene programs in the kidney. This leads to a reduction in protein levels of some, but not all mitochondrial oxidative phosphorylation components, and does not affect mitochondrial content in kidney, at least based on mtDNA levels.

### PGC-1α is important for adaptation of renal lipid metabolism with HFD feeding

To further elucidate the PGC-1α-regulated transcriptional network in kidney, we used Integrated Motif Activity Response Analysis (ISMARA) [14] to assess the core set of transcription factors (TF) that display altered activity with inactivation of PGC-1α. Interestingly, ERRα (ESRRA.p2) was amongst the top-scoring TF motifs with reduced activity in both chow-fed (S5A Fig) and HFD-fed (S5B Fig) NiPKO mice. ERRα is a well- known partner of PGC-1α in the regulation of mitochondrial gene transcription [16], and impaired ERRα activity in both chow-fed and HFD-fed NiPKO animals (S5C Fig) likely contributes to the reduction in mitochondrial gene transcription in the kidney of these mice. Additionally, in chow-fed NiPKO mice, the recently described transcriptional partner of PGC-1α/ERRα, Krüppel-like factor 4 (KLF4) [17], was predicted to have reduced activity (S5A Fig). Inversely, nuclear factor κB (NF-κB) activity (NFKB1_REL_RELA.p2) was predicted to be specifically increased in HFD-fed (S5E Fig) but not in the chow-fed (S5D Fig) NiPKO mice compared to control animals. NF-κB is a central transcriptional regulator of the inflammatory gene program, which is upregulated in kidney during high fat diet feeding (Stemmer et al., 2012). Furthermore, in skeletal muscle cells, the transcriptional activity of NF-κB and pro- inflammatory gene expression is reduced by PGC-1α [18]. Accordingly, when comparing chow-fed to HFD-fed groups, NF-κB activity was predicted to be increased with HFD feeding in NiPKO mice, but not in control mice (S3C and S3D Fig). Thus, NF-κB activity seems to be induced in NiPKO mice upon HFD-feeding to a higher extent than in control mice (S3F Fig), which could indicate that there is a difference in the inflammatory response to HFD feeding between control and NiPKO mice.

**Fig 7:**
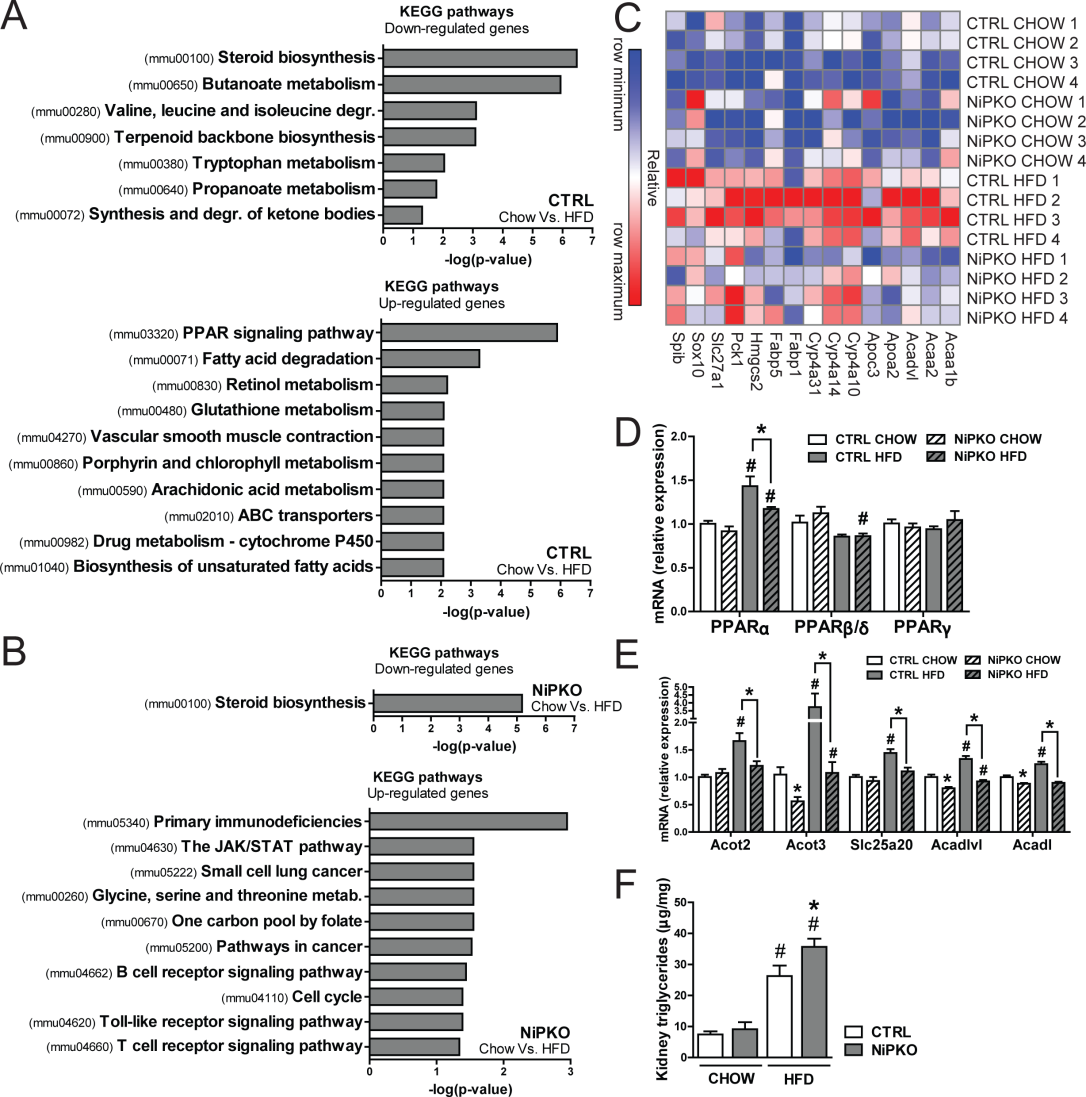
NiPKO mice develop exacerbated renal steatosis with high fat diet-feeding. KEGG pathway enrichment analysis performed on gene expression array data from kidney from either CHOW or HFD-fed control (CTRL) or NiPKO mice. (A-B) Top significant terms for KEGG pathway enrichment analysis of transcripts either down- or up-regulated with HFD-feeding in (A) CTRL or (B) NiPKO mice. (C) Heat map generated using probe set intensities for transcripts associated with the KEGG-categories “*PPAR signaling pathway*” and “*Fatty acid degradation*” in figure A, for CHOW- and HFD-fed CTRL or NiPKO mice. Row minimum = -10, row maximum = 10 fold change. (D-E) mRNA levels of indicated genes in kidney normalized to eEF2 mRNA levels, in CHOW or HFD-fed CTRL or NiPKO mice (n = 7–8). (F) Triglycerides in kidney from CHOW- and HFD-fed CTRL or NiPKO mice (n = 6–8). Error bars represent mean ±SEM. Significant differences (p-value <0.05) between genotypes are indicated by an asterisk (*) and between chow- and HFD-fed groups by a number sign (#).

Next, we analyzed the transcriptional networks affected by HFD feeding in the kidney of both genotypes via KEGG pathway enrichment analysis to investigate whether NiPKO mice display any alterations in their adaptation to a HFD compared to control mice. In regard to transcripts significantly down-regulated by HFD feeding, the term *steroid biosynthesis (mmu00100)* was significantly enriched in both NiPKO and control mice (Fig 7A and 7B, S5 Table). This indicates that this process is downregulated by HFD feeding in the kidney, but not further affected by the absence of PGC-1α. However, in line with the predicted increase in NF-κB activity, HFD-fed NiPKO mice displayed several KEGG terms enriched amongst their upregulated genes that were associated with inflammation and immune response, such as *primary immunodeficiencies (mmu05340)*, *the JAK/STAT pathway (mmu04630)* and *Toll-like receptor signaling pathway (mmu04620)* (Fig 7B, S5 Table). Importantly, none of these terms were found associated with the upregulated transcripts in HFD-fed control mice (Fig 7A, S5 Table). Contrariwise, the two most significant KEGG terms for the upregulated genes in HFD-fed control mice, *PPAR signaling pathway (mmu03320)* and *fatty acid degradation (mmu00071)* (Fig 7A), were not enriched amongst the up- regulated processes in NiPKO mice (Fig 7B). Within the KEGG-categories *PPAR signaling pathway* and *fatty acid degradation*, we found several genes associated with lipid metabolism that were induced by HFD feeding in control mice, but not in NiPKO mice (e.g. acetyl-CoA acyltransferase 2/*Acaa2*, apolipoprotein A2/*Apoa2*, fatty acid binding protein 1/*Fabp1*) (Fig 7C). Collectively, this indicates that induction of lipid metabolic processes with HFD-feeding in kidney is dependent on PGC-1α. Since one of the main upregulated terms in HFD-fed control mice was *PPAR signaling pathway*, we measured transcript levels of the peroxisome proliferator-activated receptors (PPAR) in kidney of control and NiPKO mice. Interestingly, while neither PPARβ/δ nor PPARγ were induced, PPARα transcript levels were increased in the kidney of HFD-fed mice (Fig 7D) and this induction of PPARα was blunted in HFD- fed NiPKO mice (Fig 7D). Accordingly, a similar transcriptional pattern could be observed for several PPARα target genes involved in fatty acid metabolism (Fig 7E), since HFD feeding led to an increased transcription of acyl-CoA thioesterase 2 (*Acot2*), *Acot3*, solute carrier family 25 (carnitine/acylcarnitine translocase) member 20 (*Slc25a20*), acyl-CoA dehydrogenase very long chain (*Acadvl*) and acyl-CoA dehydrogenase long chain (*Acadl*) in kidneys of control mice, and this induction was again blunted in NiPKO mice (Fig 7E). Importantly, triglyceride levels were significantly elevated in kidney of HFD-fed NiPKO mice compared to control mice (Fig 7F) indicating that the reduced transcription of fatty acid metabolism in kidney of NiPKO mice was indeed associated with alterations in renal lipid handling. Thus, PGC-1α is important for the adaptation of renal lipid metabolism in response to HFD feeding, and inactivation of PGC-1α in the kidney exacerbates renal hypertriglyceridemia and subsequent inflammation during HFD feeding.

## Discussion

The kidney has a high energy demand and relies almost exclusively on oxidative phosphorylation for ATP production [2,3]. The importance of mitochondria for renal function is underscored by the prevalence of renal dysfunction in mitochondrial cytopathies [2,4,5]. Additionally, mitochondrial dysfunction is a hallmark sign of several renal disorders [19,20,21,22]. PGC-1α is a key regulator of mitochondrial oxidative phosphorylation and oxidative metabolism [6]. Surprisingly, despite the importance of mitochondrial energy metabolism for renal function, relatively little is known about how PGC-1α influences renal function and physiology. We now demonstrate that PGC-1α is an important transcriptional regulator of mitochondrial gene programs in kidney, such as oxidative phosphorylation, TCA cycle and fatty acid metabolism. However, despite the role of PGC-1α in transcriptional regulation of mitochondrial genes, inactivation of PGC-1α in the kidney had only minor effects on the regulation of blood pressure and salt- and water homeostasis. Notably, NiPKO mice displayed a mild loss of sodium in the urine, and this phenotype was exacerbated in aged NiPKO mice upon dietary sodium restriction. Aging is associated with a reduced ability to retain sodium during salt depletion in humans [23], and with reduced renal PGC-1α levels in mice [24]. Thus, our study provides a potential link between reduced PGC-1α levels and the reduced capacity to reabsorb sodium with age. While the underlying mechanism(s) needs to be elucidated, we can demonstrate that inactivation of PGC-1α in kidney leads to decreased protein levels of the sodium transporters NKCC2 and NCCT in kidney during low-salt diet feeding. Even though the phosphorylation status of these two proteins remains unchanged, reduced levels of the two important sodium channels during LSD-feeding could explain the urinary loss of sodium with ablation of renal PGC-1α. However, without a more detailed segmental study, it is unclear how PGC-1α is involved in the function of different parts of the nephron. A rudimentary expression analysis using microdissected nephron segments indicates a relatively high expression of PGC-1α in different parts of the tubulus, at least compared to transcript levels in the glomerulus (S6 Fig). Intriguingly, a gradient towards increasing expression of PGC-1α in the distal direction of the tubulus is observed, which could be linked to the salt-losing phenotype in the knockout mice.

The importance of renal PGC-1α for the transcriptional regulation of mitochondrial and metabolic processes in kidney is in agreement with the effect of PGC-1α in other organs, such as brown adipose tissue, skeletal muscle and heart [25]. Importantly, the similarities in transcriptional profiles between these tissues could result not only from high basal expression of PGC-1α, but could also be due to the expression of the same TF partners of PGC-1α relevant for mitochondrial transcription, such as nuclear respiratory factor 1 (NRF1), mitochondrial transcription factor A (TFAM) and ERRα. Indeed, renal ERRα levels equal those of brown adipose tissue and heart and exceed the levels in skeletal muscle [26,27]. Based on motif activity response analysis of our gene expression data, we predicted a significant blunting of ERRα transcriptional activity in our NiPKO mice. ERRα is an established partner of PGC-1α in the regulation of mitochondrial gene transcription [16], and reduced activity ERRα correlates with the impaired transcription of mitochondrial genes when PGC-1α is ablated in kidney. We also predicted a significant reduction of KLF4 transcriptional activity in kidney of NiPKO mice. KLF4 was recently shown to be an essential component of the transcription of mitochondrial genes via PGC-1α and ERRα in heart [17]. Thus, KLF4 could likewise be a novel regulator of mitochondrial gene transcription working together with PGC-1α in the kidney. Furthermore, in our analysis, nuclear receptor 5A1 (NR5A1) and zinc finger protein 143 (ZNF143) were predicted to have a reduced transcriptional activity in NiPKO mice, and these TFs have been either shown or at least predicted to be transcriptional partners of PGC-1α [28,29]. Interestingly, ISMARA analysis implies both NR5A1 and ZNF143 to regulate mitochondrial gene transcription in our dataset (data not shown). However, further studies are needed to elucidate the potential role of these TFs as transcriptional partners of PGC-1α-mediated mitochondrial gene transcription in kidney.

Mitochondrial dysfunction and decreased ATP production are associated with reduced renal function and impaired transtubular transport [4,5]. Despite the inactivation of PGC-1α in kidney and the reduction in mitochondrial transcription, NiPKO mice displayed a largely normal renal phenotype. It is important to note that the effect of PGC-1α inactivation on mitochondrial protein content in our study was moderate, which in turn could explain the mild phenotype. One likely explanation for this could be that the related family member PGC-1β could compensate for the loss of PGC-1α in NiPKO mice, especially since redundancy between PGC-1α and PGC-1β on mitochondrial gene transcription has been previously shown in skeletal muscle [30]. Another explanation for this observation would be that the role of PGC-1α in kidney is more prominent during states of increased metabolic stress, as we demonstrated in the context of HFD feeding, but also in other renal disease states. Indeed, several etiologically distinct renal disorders, such as diabetic nephropathy, ischemia/reperfusion injury and sepsis are associated with reduced mitochondrial function [19,20,21,22] and reduced levels or activity of PGC-1α in the kidney [22,31,32]. The link between PGC-1α and renal disease was recently explored in two studies by Tran et al. [22,33], where the authors show that deletion of PGC-1α specifically in the renal proximal tubules leads to a worsened renal phenotype in mice during acute ischemic kidney injury [22] while overexpression of PGC-1α helps to protect against damage [33]. Recent studies have also shown that treatment with the SIRT1-activator SRT1720 [20] or calorie restriction [31], which are linked to increased PGC-1α activation, improves the renal mitochondrial phenotype and reduces ischemia/reperfusion injury in mice. Moreover, overexpression of PGC-1α in cultured proximal tubule cells protects against both TNFα- [22] and aldosterone-induced [32] mitochondrial dysfunction. Hence, while our current study demonstrates a minor role for PGC-1α in basal salt- and water handling and blood pressure regulation in kidney, increased PGC-1α activity could be a valid therapeutic strategy to ameliorate renal mitochondrial dysfunction in a broad spectrum of renal disorders. In particular, our data highlight the importance of PGC-1α in the kidney during metabolic stress, since we found that absence of PGC-1α leads to enhanced deposition of triglycerides in kidney of HFD-fed mice. Transcriptional analysis showed that upon HFD feeding, induction of PPARα and several PPARα-targets involved in fatty acid metabolism were significantly blunted in the kidney of NiPKO mice. Indeed, PPARα is a well-known transcriptional partner of PGC-1α for the regulation of lipid metabolism [34]. Importantly, ablation of PPARα in mice leads to an increased susceptibility to diabetic nephropathy [35] and exacerbated free fatty acid-induced injury in the kidney [36]. Moreover, increased PPARα-activity in obese rodents protects against renal lipotoxicity [37,38] and has been associated with increased levels of PGC-1α in kidney [39]. Obesity and HFD feeding lead to ectopic deposition of triglyceride in the kidney [38,40]. Both PGC-1α and PPARα have been described as inhibitors of NF-κB activity [18,41]. Thus, both PGC-1α and PPARα could be instrumental in attenuating an inflammatory reaction triggered by pathological accumulation of lipids in the kidney as in other tissues [42]. Our findings highlight an important link between reduced renal PGC-1α levels and deregulated lipid metabolism in kidney. Further studies are now needed to address the potential protective effect of increased PGC-1α activity in diseases associated with defective renal lipid metabolism and lipotoxicity.

In summary, we found that PGC-1α plays a marked role in the transcriptional regulation of several interconnected mitochondrial gene programs in the kidney, such as oxidative phosphorylation, TCA cycle and fatty acid metabolism. While inactivation of tubular PGC-1α resulted in a minor increase in urinary sodium excretion that was exacerbated by age and high fat diet feeding, there was no further impairment in salt- and water homeostasis or blood pressure regulation in NiPKO mice. Importantly, our data demonstrate a crucial role for PGC-1α in the regulation of renal lipid metabolism during metabolic stress, leading to elevated renal steatosis in the absence of functional PGC-1α. While our findings point towards a minor role of PGC-1α in basal renal physiology, we thus hypothesize that activation of PGC-1α in the context of renal disorders could be a valid therapeutic strategy to ameliorate renal mitochondrial dysfunction in a broad spectrum of renal disorders.

## Supporting Information

S1 Fig Histological kidney sections. A-B) Representative PAS staining of kidney regions or whole kidney sections at 1 and 12 months after doxycycline administration (n = 3).
(PDF)

S2 Fig Plasma ion levels and ISMARA prediction. (A-B) Plasma levels of calcium (Ca^2+^), chloride (Cl^-^), potassium (K^+^), sodium (Na^+^), protein, urea at (A) 1 month and (B) 12 months after doxycycline administration (n = 5–12). (C-D) Transcription factor motifs predicted to have altered activity in ISMARA analysis of CHOW-fed and HFD-fed (C) control and (D) NiPKO mice. Error bars represent mean ±SEM. Significant differences (p-value<0.05) between genotypes are indicated by an asterisk (*).
(PDF)

S3 Fig Food intake. Food intake in Control and NiPKO mice. Food intake was measured for individually-housed mice in metabolic cages on 5 consecutive days and normalized for intake over 24 hours.
(PDF)

S4 Fig Transporter gene expression analysis. mRNA levels of indicated genes in kidney normalized to *eEF2* mRNA levels, in either CHOW- or HFD- fed control (CTRL) or NiPKO mice (n = 7–8). Error bars represent mean ±SEM. Significant differences (p-value <0.05) between genotypes are indicated by an asterisk (*) and between chow- and HFD-fed groups by a number sign (#).
(PDF)

S5 Fig Motif activity prediction. A-B) Transcription factor (TF) motifs predicted to have decreased activity in ISMARA analysis between (A) CHOW-fed control and NiPKO mice or (B) HFD-fed control and NiPKO mice. (C) Changes in ERRα (ESRRA.p2) activity in kidney from either chow or HFD-fed CTRL or NiPKO mice, as predicted by ISMARA analysis. (D-E) Transcription factor (TF) motifs showing increased activity in ISMARA analysis between (D) CHOW-fed control and NiPKO or (E) HFD-fed control and NiPKO mice. (F) Changes in NF-κB (NFKB1_REL_RELA.p2) activity in kidney from either chow or HFD-fed CTRL or NiPKO mice, as predicted by ISMARA analysis.
(PDF)

S6 Fig Expression of PGC-1α in different kidney segments. Expression of PGC-1α was measured from RNA extracted from microdissected nephron segments and glomeruli. PGC-1α transcript levels are normalized to TATA-binding protein gene expression and depicted according to the ΔCt method.
(PDF)

S1 Table Primer sequences. qPCR primer sequences.
(PDF)

S2 Table List of down-regulated genes. List of genes significantly down-regulated (p<0.05, FC<1.2) between chow-fed control (CWT), chow-fed NiPKO (CKO), HFD-fed control (HWT) and HFD-fed NiPKO (HKO) groups. The heading “Groups” lists in which comparison the relevant gene(s) are found to be down-regulated.
(PDF)

S3 Table List of up-regulated genes. List of genes significantly up-regulated (p<0.05, FC<1.2) between chow-fed control (CWT), chow-fed NiPKO (CKO), HFD-fed control (HWT) and HFD-fed NiPKO (HKO) groups. The heading “Groups” lists in which comparison the relevant gene(s) are found to be up-regulated.
(PDF)

S4 Table Functional analysis of gene expression in chow-fed mice. Genes associated with KEGG pathways in CTRL vs. NiPKO animals.
(PDF)

S5 Table Functional analysis of gene expression in HFD-fed mice. Genes associated with KEGG pathways CHOW vs. HFD treated animals.
(PDF)

## Abbreviations

DOX: doxycycline
HFD: high fat diet
ISMARA: Integrated Motif Activity Response Analysis
LSD: low salt diet
mtDNA: mitochondrial DNA
NiPKO: nephron-specific inducible PGC-1α knockout
PGC-1α: peroxisome proliferator-activated receptor γ coactivator 1α
PPAR: peroxisome proliferator-activated receptor
rtTA: reverse tetracycline-controlled transactivator
TF: transcription factor
