# Supplementary figures and images for: Loss of Renal Tubular PGC-1α Exacerbates Diet-Induced Renal Steatosis and Age-Related Urinary Sodium Excretion in Mice
Source: PLoS One. 2016 Jul 27;11(7):e0158716. doi: 10.1371/journal.pone.0158716 (PMC4963111; doi:10.1371/journal.pone.0158716)

**A**

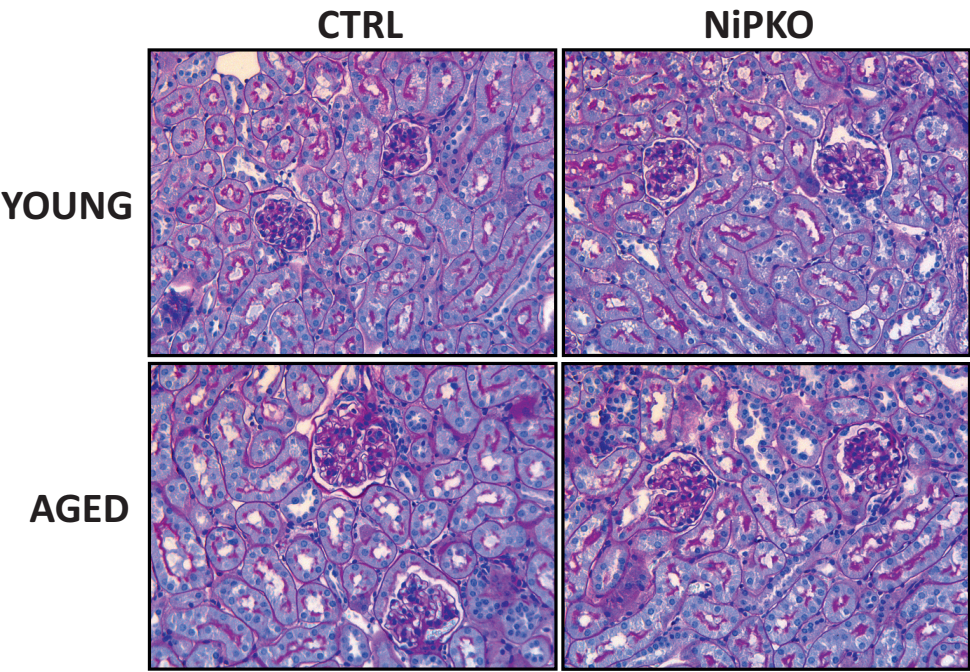

**B**

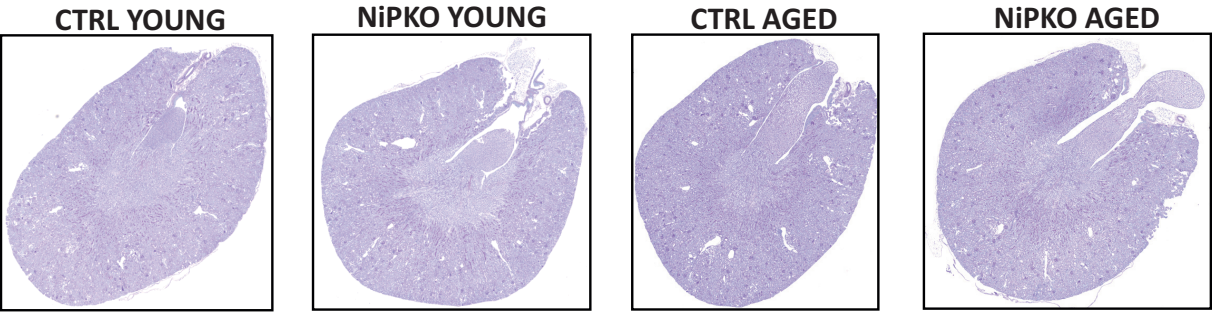

Supplement: S1 Fig — A-B) Representative PAS staining of kidney regions or whole kidney sections at 1 and 12 months after doxycycline administration (n = 3). (PDF) [file pone.0158716.s001.pdf]

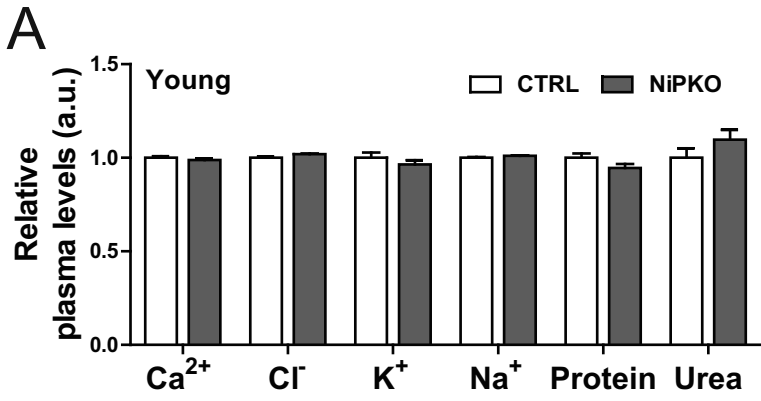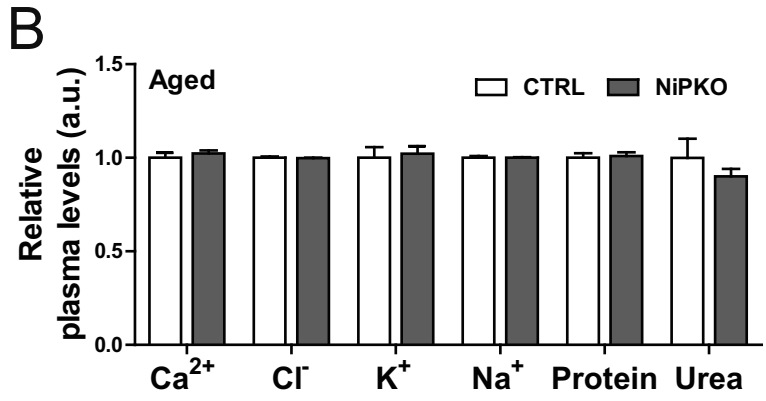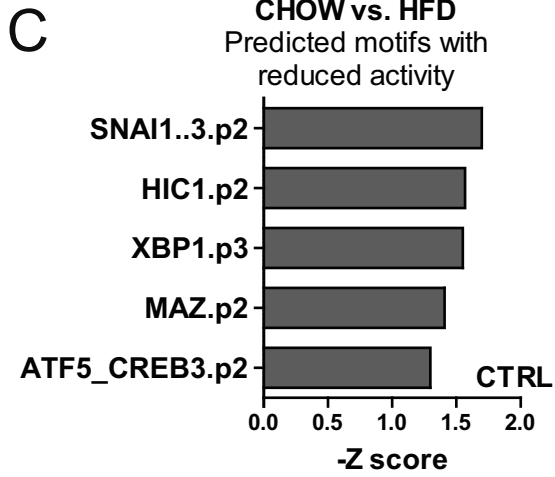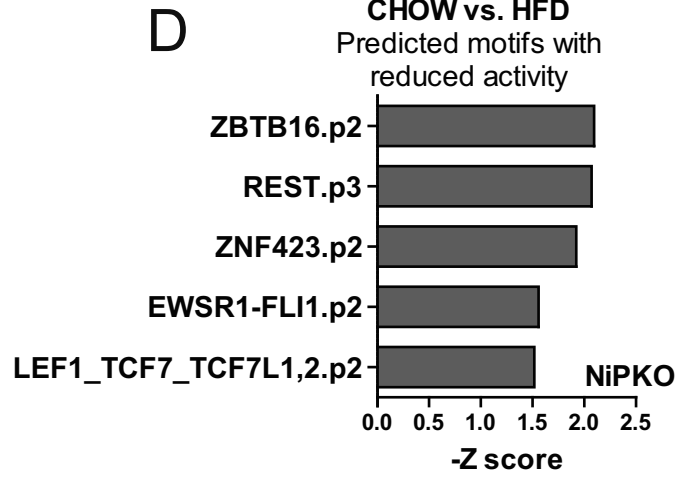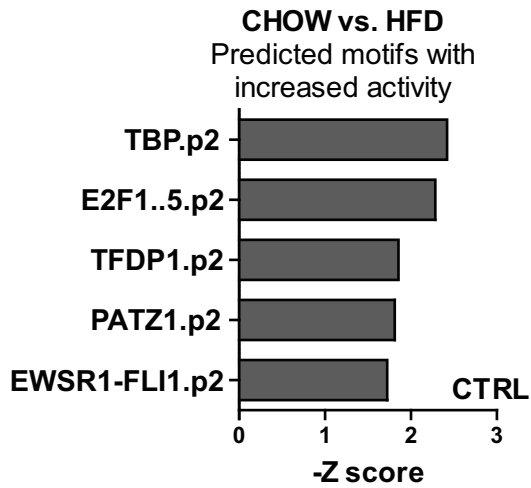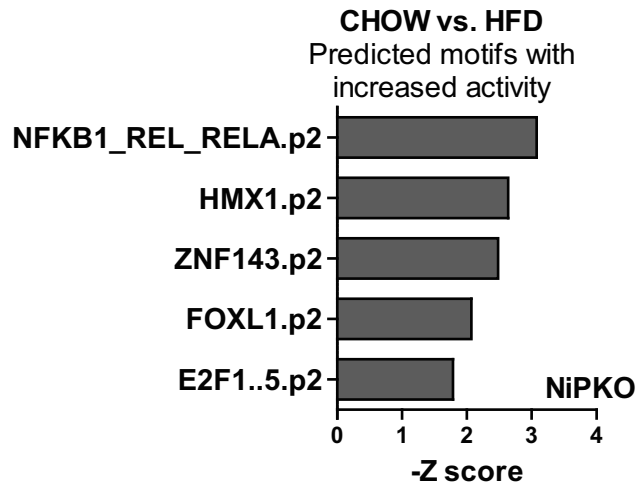

Supplement: S2 Fig — (A-B) Plasma levels of calcium (Ca2+), chloride (Cl-), potassium (K+), sodium (Na+), protein, urea at (A) 1 month and (B) 12 months after doxycycline administration (n = 5–12). (C-D) Transcription factor motifs predicted to have altered activity in ISMARA analysis of CHOW-fed and HFD-fed (C) control and (D) NiPKO mice. Error bars represent mean ±SEM. Significant differences (p-value<0.05) between genotypes are indicated by an asterisk (*). (PDF) [file pone.0158716.s002.pdf]

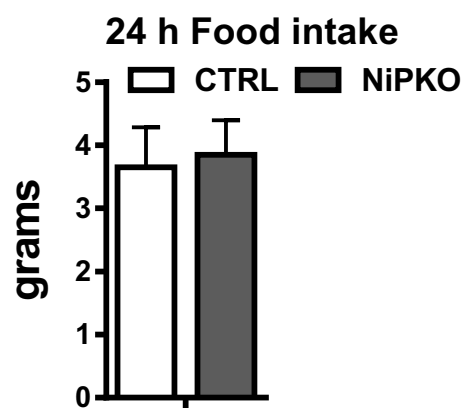

Supplement: S3 Fig — Food intake in Control and NiPKO mice. Food intake was measured for individually-housed mice in metabolic cages on 5 consecutive days and normalized for intake over 24 hours. (PDF) [file pone.0158716.s003.pdf]

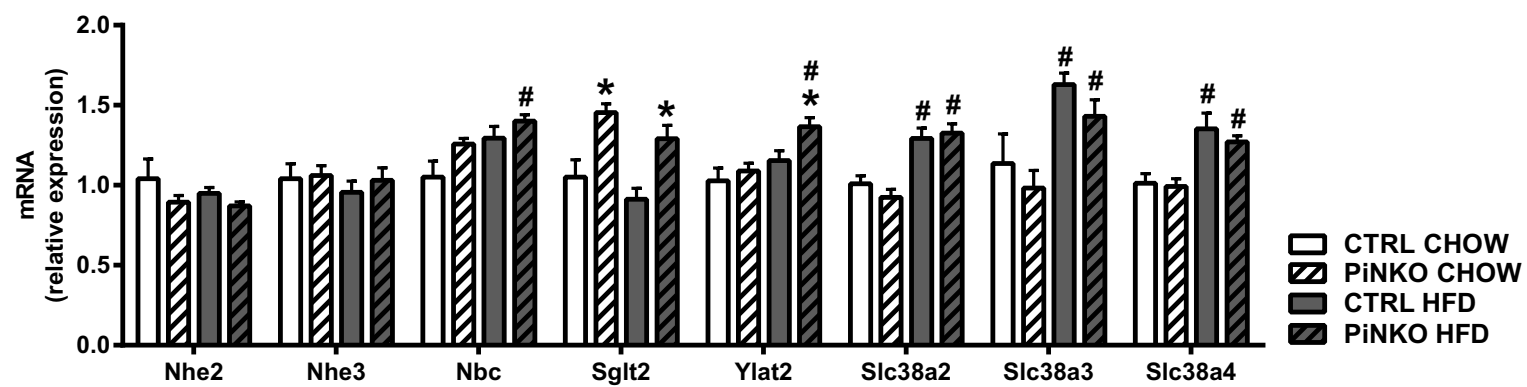

Supplement: S4 Fig — mRNA levels of indicated genes in kidney normalized to eEF2 mRNA levels, in either CHOW- or HFD- fed control (CTRL) or NiPKO mice (n = 7–8). Error bars represent mean ±SEM. Significant differences (p-value <0.05) between genotypes are indicated by an asterisk (*) and between chow- and HFD-fed groups by a number sign (#). (PDF) [file pone.0158716.s004.pdf]

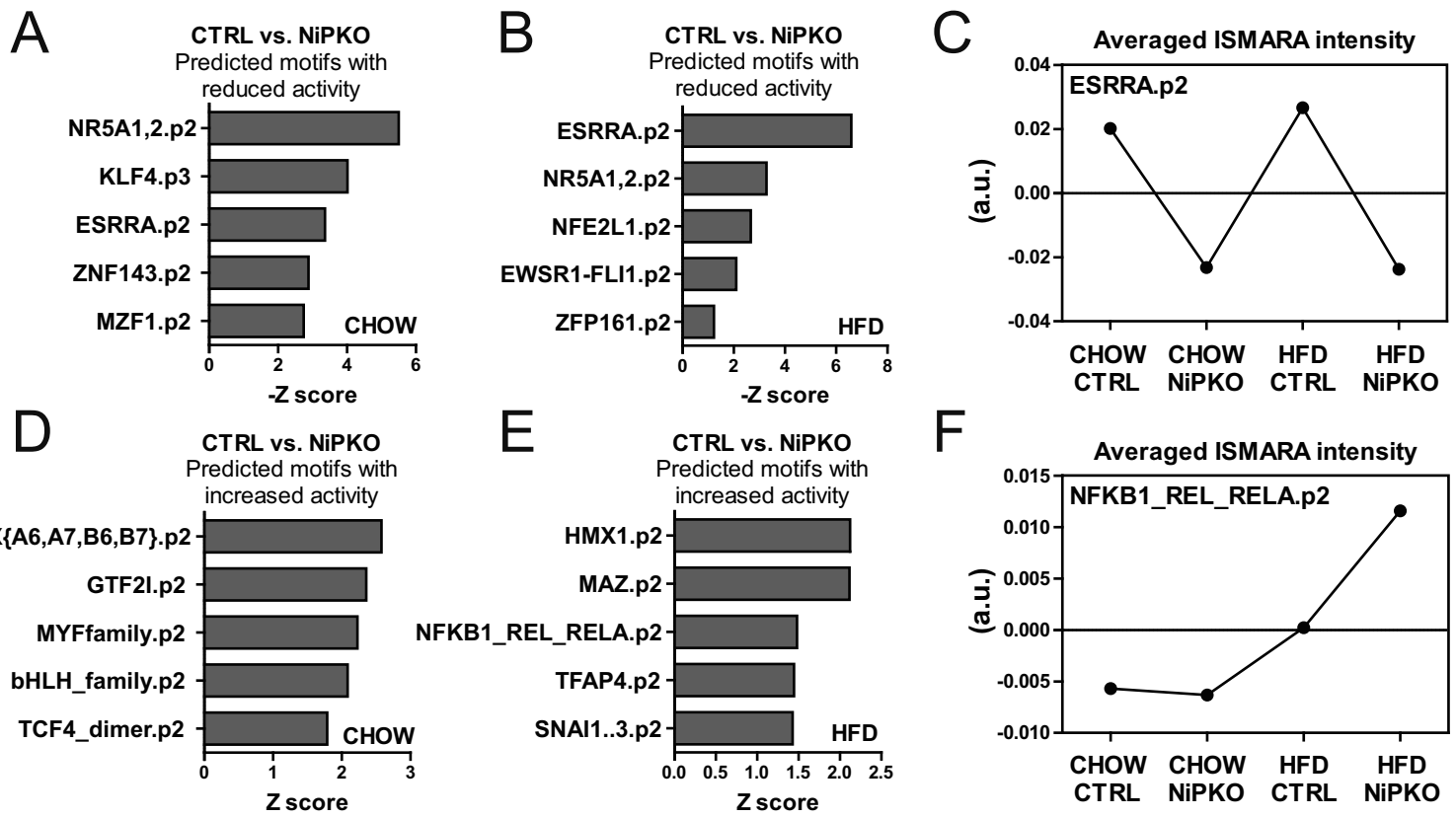

S5 Fig

Supplement: S5 Fig — A-B) Transcription factor (TF) motifs predicted to have decreased activity in ISMARA analysis between (A) CHOW-fed control and NiPKO mice or (B) HFD-fed control and NiPKO mice. (C) Changes in ERRα (ESRRA.p2) activity in kidney from either chow or HFD-fed CTRL or NiPKO mice, as predicted by ISMARA analysis. (D-E) Transcription factor (TF) motifs showing increased activity in ISMARA analysis between (D) CHOW-fed control and NiPKO or (E) HFD-fed control and NiPKO mice. (F) Changes in NF-κB (NFKB1_REL_RELA.p2) activity in kidney from either chow or HFD-fed CTRL or NiPKO mice, as predicted by ISMARA analysis. (PDF) [file pone.0158716.s005.pdf]

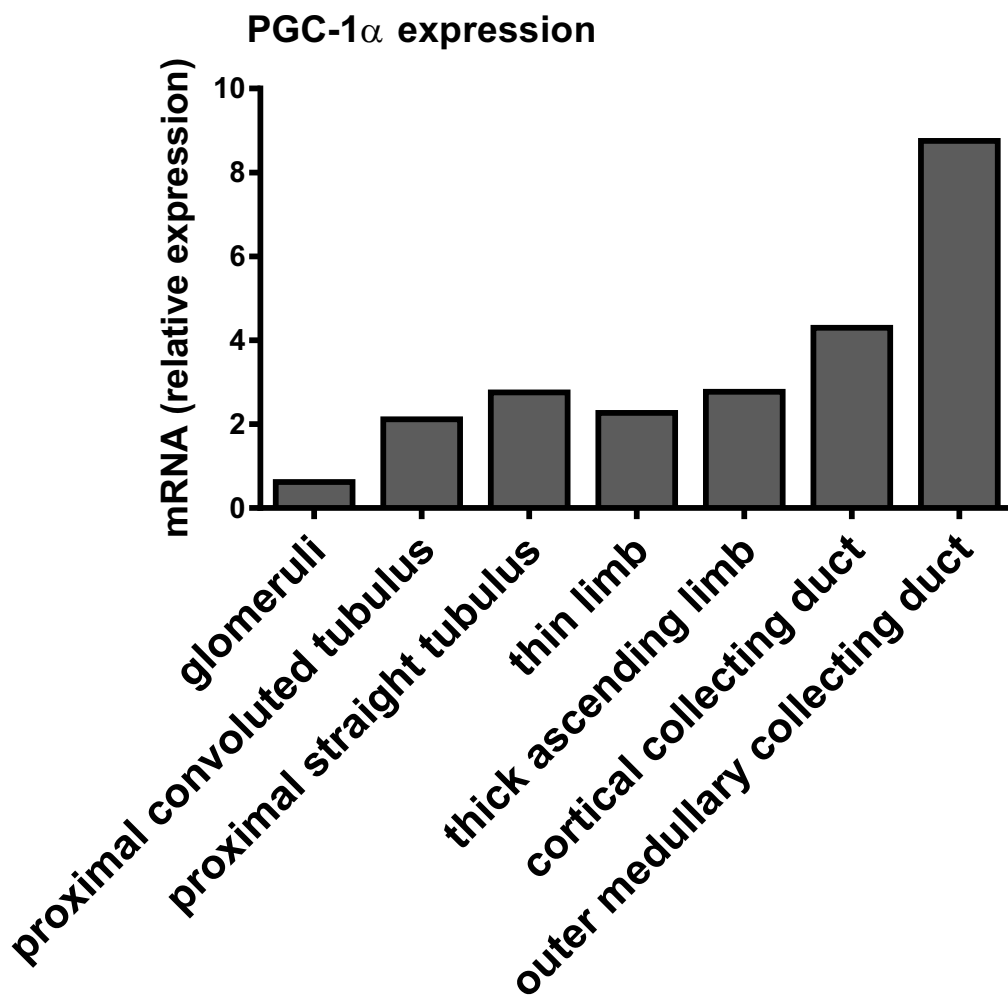

Supplement: S6 Fig — Expression of PGC-1α was measured from RNA extracted from microdissected nephron segments and glomeruli. PGC-1α transcript levels are normalized to TATA-binding protein gene expression and depicted according to the ΔCt method. (PDF) [file pone.0158716.s006.pdf]
