# Supplementary material for: Loss of Renal Tubular PGC-1α Exacerbates Diet-Induced Renal Steatosis and Age-Related Urinary Sodium Excretion in Mice
Source: PLoS One. 2016 Jul 27;11(7):e0158716. doi: 10.1371/journal.pone.0158716 (PMC4963111; doi:10.1371/journal.pone.0158716)
Supplement: S1 Table — qPCR primer sequences. (PDF) [file pone.0158716.s007.pdf]

S1 Table

Target gene	Forward primer	Reverse primer
**Acadl**	CCAGCTAATGCCTTACTTGGAGA	GCAATTAAGAGCCTTTCCTGTGG
**Acadvl**	GTAGCCTCCATCCGAAGCTC	CAGGCCCCCATTACTGATCC
**Acot2**	AGTGCCTATGAAGGACTGAGG	TCTTACGGCACTGGGGAATG
**Acot3**	AGCTCTTGACCTTGTCTGTCTG	GAGAAAGTTCAAGCAAACTGGG
**Atp5h**	GGGGGTCGGTGAAGTATCC	AATTGCCTTCTGGTTTTGGGG
**Atp5k**	GCCAAGCGCTACAGTTACCTA	GCCAGTTCTCTCTCAATCCGT
**Cox5a**	CCTGGGAATTGCGTAAAGGGA	TAACCGTCTACATGCTCGCAA
**Cs**	CCCAGGATACGGTCATGCA	GCAAACTCTCGCTGACAGGAA
**D-loop (mtDNA)**	GGTTCTTACTTCAGGGCCATCA	GATTAGACCCGTTACCATCGAGA<br>T
**eEF2**	ATACCTGCCTGTCAATGAGTCCTT	CTGGCCGCCGGTGTT
**ENaCα**	TGTGCATTCACTCCTGCTTC	ACCCTTGGGCTTAGGGTAGA
**ENaCβ**	TGGTTGATAGGCCACTTTCC	AGAGGTCAACCAATGCCAAC
**ENaCγ**	TTACAGCCAGTGCACAGAGG	AAGGCAGATCTGGAGGGAAT
**Errα**	CGGTGTGGCATCCTGTGA	CTCCCCTGGATGGTCCTCTT
**Nbc**	GAAATCCAACCTTCGGTCCCT	GGCTGCCATTATCAGGGTTG
**Ncct**	TGCTGACTTGGCTCATCATC	GACTTGACCTTGCCATTGGT
**Ndufa1**	TCCACTGCGTACATCCACAAA	CGTCTATCGCGTTCCATCAGA
**Ndufs3**	AAAGACTTTCCCCTCACTGGC	AACTTGCGGAATTCTTGTGCC
**Ndufv1**	CTTCCCCACTGGCCTCAAG	CCAAAACCCAGTGATCCAGC
**Nhe2**	CAAGTCCTGCCAGTGTGGTG	GGGGGTAATCCAGGGTGAAC
**Nhe3**	CACCTTCAAATGGCACCACG	TGTGGGACAGGTGAAAGACG
**Nkcc2**	TGGAAGCAGTCAAGGAAAAG	ACTGGAAGGCTCAACACGAT
**PGC-1α**	AGCCGTGACCACTGACAACGAG	GCTGCATGGTTCTGAGTGCTAAG
**PGC-1β**	CCATGCTGTTGATGTTCCAC	GACGACTGACAGCACTTGGA
**PPARα**	ACAAGGCCTCAGGGTACCA	GCCGAAAGAAGCCCTTACAG
**PPARβ/δ**	GCAAGCCCTTCAGTGACATCA	CCAGCGCATTGAACTTGACA
**PPARγ**	CCCACCAACTTCGGAATCAG	AATGCGAGTGGTCTTCCATCA
**PRC**	CACCCTGCCGGAGTGAAAT	CGCATTGACTGCTGCTTGTC
**Sglt2**	TTGGTGTTGGCTTGTGGTCT	GAACAGAGAGGCTCCAACCG
**Slc25a20**	CTGCGCCCATCATTGGA	CAGACCAAACCCAAAGAAGCA
**Slc38a2**	GCAGTGGAATCCTTGGGCTT	GGAGATGGACGGAGTATAGCG
**Slc38a3**	GTACGGCCGGGGGATATAGA	TCGGGGTACAAGGAGAGCTT
**Slc38a4**	AGACAGAAAGGCGGGAAAGG	CCTGAATACTGTCCCCGCTG
**Slc7a7**	AGGCTGTTAAGAGGCGGAAC	AATGGGGGTGTGACTTCAGC
**Tbp**	TGCTGTTGGTGATTGTTGGT	CTGGCTTGTGTGGGAAAGAT
**Uqrfs1**	CCTGAAGGGAAGAACATGGCT	TAACTGGGACACTTCGACTGC
**Ylat2**	GACGGCGCGACCGAG	CCTGTGCTTAGTATGGCCTCC
