# Supplementary material for: Loss of Renal Tubular PGC-1α Exacerbates Diet-Induced Renal Steatosis and Age-Related Urinary Sodium Excretion in Mice
Source: PLoS One. 2016 Jul 27;11(7):e0158716. doi: 10.1371/journal.pone.0158716 (PMC4963111; doi:10.1371/journal.pone.0158716)
Supplement: S2 Table — List of genes significantly down-regulated (p<0.05, FC<1.2) between chow-fed control (CWT), chow-fed NiPKO (CKO), HFD-fed control (HWT) and HFD-fed NiPKO (HKO) groups. The heading “Groups” lists in which comparison the relevant gene(s) are found to be down-regulated. (PDF) [file pone.0158716.s008.pdf]

S2 Table

Groups	Number of genes	Genes
**CKOxHKO<br>CWTxCKO<br>CWTxHWT<br>HWTxHKO**	**3**	Acot11<br>Dhcr7<br>Tiam2
**CWTxCKO<br>CWTxHWT<br>HWTxHKO**	**4**	LOC100862384<br>LOC100862317<br>Ndrg2<br>Oxct1
**CKOxHKO<br>CWTxCKO<br>HWTxHKO**	**1**	2610029I01Rik
**CKOxHKO<br>CWTxCKO<br>CWTxHWT**	**2**	4732415M23Rik<br>Aldh1l1
**CWTxCKO<br>HWTxHKO**	**147**	1110001J03Rik<br>1700028J19Rik<br>1700040L02Rik<br>1810014F10Rik<br>2010107E04Rik<br>2310061C15Rik<br>2410003L11Rik<br>4930452B06Rik<br>4930481A15Rik<br>Acsl3<br>Adcy1<br>Afg3l2<br>Agl<br>Agt<br>Ak1<br>Ak3<br>Akr1b10<br>Apoo<br>Arhgap6<br>Aspdh<br>Aste1<br>Atp5e<br>Atp5f1<br>Atp5g3<br>Atp5h<br>Atp5sl<br>Baiap2l2<br>BC090627<br>Bola3<br>C030037D09Rik<br>Cadm4<br>Ccdc68<br>Chchd10<br>Ckmt1<br>Coq9<br>Cox10<br>Cox5a<br>Cox7a1<br>Cox7a2<br>Cyc1<br>Cycs<br>Cyp2d22<br>Cyp2u1<br>Dlat<br>Dlst<br>Dpp4<br>Enpp6<br>Epb4.9<br>Etfdh<br>Faah<br>Fastk<br>Fastkd1<br>Fbxo18<br>Fbxo44<br>Fh1<br>Gfm1<br>Glod5<br>Glrx<br>Gm10767<br>Gm16845<br>Gm19447<br>Gm20141<br>Gm2695<br>Gm53<br>Gm906<br>Gnb5<br>Got1<br>Gsto1<br>Impa2<br>Kcna4<br>Khdrbs3<br>Klk1b5<br>Klk1b7-ps<br>Ldhb<br>LOC100046079<br>LOC100862063<br>Luzp1<br>Lynx1<br>Lyrm5<br>Mdh2<br>Mir196a-1<br>Mrpl13<br>Mrps35<br>Msrb2<br>Mtfp1<br>Ndufa1<br>Ndufa12<br>Ndufa4<br>Ndufa8<br>Ndufa9<br>Ndufaf4<br>Ndufc1<br>Ndufs3<br>Ndufs8<br>Nol3<br>Nr0b2<br>Nudt7<br>Oplah<br>Oxnad1<br>Pcolce<br>Pfkm<br>Pfkp<br>Pla2g7<br>Pon3<br>Ppif<br>Ptges2<br>Ptpn18<br>Rtn4ip1<br>Sirt3<br>Slc12a7<br>Slc16a5<br>Slc25a12<br>Slc25a4<br>Slc2a4<br>Slc6a8<br>Srxn1<br>Stard8<br>Suv420h2<br>Sv2a<br>Tarm1<br>Thap4<br>Tmem35<br>Tmem38a<br>Tom1l1<br>Trmt2b<br>Trp53inp2<br>Tspo<br>Ubac1<br>Uqcr10<br>Uqcrb<br>Uqcrc1<br>Uqcrfs1<br>Uqcrh<br>Uqcrq<br>Vmn1r157<br>Zdhhc23

		Clybl	Gys1	Pgam1	Zfp30
		Cntnap5b	Hsdl2	Pgk1	Zfp691
		Comtd1	Idh3a	Pigy	
**CWTxCKO CWTxHWT**	**48**	1110058L19Rik	Cyp2c29	Gm7429	Mup-ps12
		1700040F15Rik	Cyp2c40	Gpx6	Olfr490
		4933409K07Rik	Echs1	Hist1h4i	Olfr851
		4933413C19Rik	Ehd1	Hspa8	Omd
		A230107N01Rik	Ftl1	Ipp	Pm20d2
		Acot9	Gbp11	Klhl3	Prl2c5
		AI854703	Gm17361	Klrd1	Slc25a19
		Aqp3	Gm20066	LOC100861804	Smcr7
		Arhgef19	Gm20251	LOC100861972	Spr
		Cd93	Gm2427	Mir140	Tbc1d24
		Chka	Gm5039	Mir151	Tgoln1
		Cyp26b1	Gm5347	Mmp13	Tmem136
**CKOxHKO HWTxHKO**	**42**	1700042G15Rik	Efcab4b	Igfbp1	Them4
		2610042L04Rik	Fgf5	Lin28a	Timm9
		4930412M03Rik	G630093K05Rik	Mir344d-2	Tmem202
		4931408D14Rik	G6pc	Myl6	Tmem86a
		5430421F17Rik	Gm13430	Olfr1390	Uhmk1
		Apol7c	Gm3696	Olfr467	Vmn1r39
		Cdh18	Gm428	Olfr479	Wnk2
		Crabp2	Gm7710	Olfr700	Zfp783
		Cyp2j8	Hbb-bh2	Osr2	Zscan4c
		Defb35	Hes2	Pax4	
		Dnajc6	I830127L07Rik	Sstr2	
**CKOxHKO CWTxHWT**	**180**	1110032F04Rik	D630022N01Rik	Igh-VJ558	S100g
		1300002K09Rik	Defa17	Insig1	Samd8
		1700113A16Rik	Defb19	Itgb6	Sc4mol
		1810034E14Rik	Defb29	Itih2	Scd1
		2310003L06Rik	Depdc1b	Itih5	Sec14l3
		2310030G06Rik	Dhcr24	Ivd	Sectm1b
		3110062M04Rik	Dpy19l3	Kcng1	Sesn2
		8430408G22Rik	Eml5	Kif20b	Slc22a19
		9030619P08Rik	Erc2	Klf15	Slc22a7
		Aadat	Fads1	Klk1b11	Slc25a48
		Abat	Fam55c	Klk1b16	Slc26a1
		Ace	Fasn	Ldhd	Slc35d2
		Acox2	Fbxl2	Ldlr	Slc41a2
		Acsm1	Fdft1	Lpl	Slc6a15
		Acsm3	Fdps	Lrrc31	Slc7a4
		Acsm5	Fmo5	Ly6f	Slc8a1
		Adh6-ps1	Fn1	Mas1	Slc9a8
		Agps	Galns	Med14	Slco1a1
		Akr1c14	Galnt1	Mep1b	Smcp
		Akr1c18	Ggct	Mfap3l	Snx31
		Amacr	Glt1d1	Mpv17l	Sqle
		Angptl7	Gm11827	Mrpl54	Srebf2
		Anks1b	Gm128	Myo5a	St8sia1
		Anxa13	Gm17767	Naaladl2	Sucnr1
		Arsb	Gm19648	Neurog2	Tbc1d10a
		Atp11a	Gm19963	Nlrc4	Tc2n

		Atp8b4	Gm4285	Npas2	Tfpi2
		B4galt5	Gm6300	Nsdhl	Timd2
		Bcmo1	Gm853	Nt5e	Tm7sf2
		Bdh1	Gnmt	Odc1	Tmed6
		C1qtnf3	Gpt	Olfr153	Tmem237
		C1rl	Gstm7	Osbp2	Tmem26
		C7	Gstt2	Pacrg	Tmem45b
		Calb1	Gusb	Pde6a	Tmem97
		Car4	H2-Q10	Pelo	Tmprss9
		Casc4	Hbegf	Pion	Treh
		Cblc	Hpd	Plau	Tspan13
		Cbs	Hsd11b1	Podn	Tspan5
		Ccbl2	Hsd17b11	Proc	Tstd1
		Ccl28	Hsd17b2	Proz	Ttc39c
		Cdhr2	Hsd3b2	Ptpn22	Ttr
		Cndp2	Hsf2bp	Rab20	Ubiad1
		Coasy	Idi1	Rab27a	Veph1
		Cpe	Ido2	Rhoc	Wfdc16
		Cryaa	Igf2bp1	Rundc3a	Zbtb44
**CWTxCKO**	**297**	0610012G03Rik	Foxred1	Mir677	Sdha
		0610038B21Rik	Fpr-rs3	Mkks	Sdhc
		1520402A15Rik	Fst	Mmachc	Sdhd
		1700034P13Rik	Fv1	Mpv17l2	Serf2
		1810014B01Rik	Gadd45gip1	Mrpl34	Sfxn3
		1810021B22Rik	Gas6	Mrpl47	Shmt1
		2310003L22Rik	Gbas	Mrps23	Skil
		2310042D19Rik	Gca	Mrps25	Slc19a2
		2310061I04Rik	Gja1	Mrps27	Slc1a5
		2410015M20Rik	Glul	Mtrf1l	Slc22a4
		2610017I09Rik	Gm10589	Mtx2	Slc43a2
		2810454H06Rik	Gm13288	Mtx3	Slc45a3
		4930402H24Rik	Gm14057	Mug1	Slc4a8
		4930461G14Rik	Gm14379	Myl12a	Slit2
		4930480K23Rik	Gm19265	Myl6b	Snora2b
		4933401B06Rik	Gm20186	Myo19	Snora34
		6430590A07Rik	Gm3613	ND6	Snord11
		9130230N09Rik	Gm4567	Ndrg1	Snord12
		A330021E22Rik	Gm4759	Ndufa6	Snord1b
		A630020A06	Gm5113	Ndufb2	Snord42b
		Abcb9	Gm561	Ndufb5	Snord58b
		Abhd11	Gm7609	Ndufb8	Snord61
		Actg1	Gm8842	Ndufs1	Snord68
		Adssl1	Gm8995	Ndufs2	Sp100
		AK129341	Gm9833	Ndufs6	Spa17
		Akr1c20	Gm9917	Ndufv2	Spata17
		Alkbh7	Gna14	Nfyb	Srp54c
		Asap2	Gnb2l1	Nipsnap1	St6galnac5
		Aspn	Gpt2	Nkap	Stc1
		Atg4d	Hadhb	Nmnat1	Stra6
		Atp10d	Hdhd2	Olfm4	Syne1
		Atp2b4	Hist2h2ab	Olfr113	Synj2bp
		Atp4a	Hk1	Olfr1135	Syt17
		Atp5d	Idh3b	Olfr1137	Tarsl2

		Atp5l	Il15ra	Olfr1278	Tas2r131
		B230369F24Rik	Inpp5j	Olfr1445	Tceb1
		BC002163	Irs1	Olfr1501	Tfb2m
		BC037704	Isyna1	Olfr342	Tfrc
		Bcar3	Jrkl	Olfr51	Ticam1
		Bcr	Jun	Olfr74	Tie1
		Blcap	Kansl2	Olfr866	Tmco4
		Bri3bp	Kansl3	Olfr945	Tmem143
		Cd200	Kcnj2	P2ry1	Tmem241
		Cd38	Klhdc8a	Paqr5	Tmem69
		Cdc14b	Klk1b22	Pclo	Tomm5
		Cfhr2	Ksr2	Pdgfd	Tsen54
		Chml	Lace1	Pdha1	Tspan1
		Clca1	Lad1	Pdhb	Ttc19
		Clec4b1	LOC100048410	Pdss2	Ttc30a2
		Cnih4	LOC100861803	Pdxk	Ube2a
		Cnnm4	LOC100862007	Pgam2	Vdac2
		Cntfr	LOC100862061	Pi4kb	Vdac3
		Cox4i1	Lrig1	Pnpla1	Vipar
		Cox7b	Lrp12	Polr2h	Vmn2r17
		Cpeb3	Lrrc66	Ppan	Vps13d
		Cs	Lyrm7	Ppm1k	Wbscr16
		Csprs	Lzic	Pptc7	Wdr72
		Cst3	Macrod1	Prdx2	Wfdc15b
		Cuedc1	Malat1	Prom2	Xirp2
		D5Ertd579e	Mapk4	Prr5	Xrcc6bp1
		D630041G03Rik	Mavs	Ptgr2	Zdhhc18
		D730048I06Rik	Mcee	Ptpru	Zdhhc8
		Dbn1	Mcpt4	Pygm	Zfp456
		Ddo	Me3	Rab6b	Zfp52
		Dennd2d	Meaf6	Rabggtb	Zfp534
		Dennd5b	Mir181a-2	Rad1	Zfp606
		Dhrs11	Mir1933	Rarb	Zfp780b
		Dhrs3	Mir1941	Ripply3	Zfp944
		Dld	Mir1949	Rmnd1	Zfp953
		Dnajc28	Mir218-1	Rnmtl1	Zfp955b
		Dnase1	Mir27b	Rny1	Zfy2
		Dusp15	Mir30b	Rtp3	Zxdb
		Etfa	Mir30c-1	Rusc1	
		Fam164c	Mir30c-2	Samm50	
		Fam73b	Mir30e	Sco1	
**HWTxHKO**	**212**	1300001I01Rik	Csrp2	Gm7367	Olfr890
		1700023F06Rik	Cux2	Gm7732	Olfr943
		1700025D23Rik	Cyp2d13	Gm8777	Oog2
		1810013D10Rik	Cyp46a1	Gm904	Pacsin1
		2300002M23Rik	D0H4S114	Gm9442	Pcdhb7
		2310046A06Rik	D630042P16Rik	Gpc1	Pgls
		2310068J16Rik	Dcaf4	Gpr162	Pla2g16
		2500002B13Rik	Defa-ps1	Gpx2	Ppcs
		3110047P20Rik	Dhrs2	Grin2d	Ppm1l
		4921509C19Rik	Dnahc7b	Grsf1	Prokr2
		4922502N22Rik	Dub2a	Guca1b	Psg-ps1
		4930444G20Rik	E130306D19Rik	H2-M10.3	Ptges3l

		4930478L05Rik	Efna2	Hadha	Pttg1
		4930503H13Rik	Efs	Hddc3	Rab30
		4930524N10Rik	Esrra	Hist1h3f	Retsat
		4933407G14Rik	Expi	Hlx	Rhox4f
		5730416F02Rik	Fam5b	Hsd17b14	Rnu11
		5830473C10Rik	Fchsd1	Ifitm6	Rtkn2
		9330159M07Rik	Fdx1	Il25	Sbk1
		9430037G07Rik	Fgf6	Kcna7	Sema5b
		9530046B11Rik	Fn3k	Kif15	Serpinb1a
		Abhd8	Ftcd	Klhl34	Slc22a20
		Abtb2	Fxn	Klrk1	Slc25a11
		Acaa2	G6pd2	Krtap8-1	Slc25a20
		Acacb	Gapdh	Ldlrad2	Slc25a22
		Acadl	Gbe1	LOC100862601	Slc38a1
		Acadvl	Gckr	LOC626410	Slc41a3
		Acot2	Gimap1	Lrrc61	Slc5a10
		Acot3	Gm10081	Mageb16	Slc5a11
		Aim1l	Gm10696	Mir1839	Slc6a17
		Als2cl	Gm11937	Mir210	Spata21
		Alyref2	Gm12302	Mir299	Spp1
		Ankrd34b	Gm12356	Mir466g	Ssxb1
		Apoa2	Gm12603	Mir540	Tm6sf2
		Arc	Gm12830	Mrpl10	Tmem236
		Atp2a1	Gm13369	Myod1	Tmie
		Atp5c1	Gm1679	Nefl	Tpi1
		Atp5g1	Gm19304	Nmur1	Uqcr11
		Atp5k	Gm19484	Nmur2	Vegfb
		AY358078	Gm19951	Nr1i2	Vmn1r188
		B3gnt3	Gm20187	Nrg3	Vmn1r2
		BC018473	Gm221	Olfr103	Vmn1r224
		BC048609	Gm2897	Olfr109	Vmn1r58
		C2cd4a	Gm3099	Olfr1167	Vmn2r20
		Ccnyl1	Gm3500	Olfr1347	Vmn2r51
		Chchd2	Gm3591	Olfr429	Vmn2r93
		Chrm1	Gm5114	Olfr513	Wfdc9
		Chrng	Gm5237	Olfr574	Wnt8a
		Clic6	Gm5523	Olfr622	Wnt8b
		Cnr1	Gm5567	Olfr63	Ybx2
		Cox5b	Gm5785	Olfr853	Zfp352
		Crat	Gm6086	Olfr856-ps1	Zfp629
		Creb3l3	Gm732	Olfr884	Zfp704
**CWTxHWT**	**308**	1110002B05Rik	Cyp7b1	Iigp1	Pzp
		1110025L11Rik	D130020L05Rik	Ipmk	Rab32
		1190003J15Rik	D630024D03Rik	Irx3	Ralgps2
		2010012O05Rik	Dcn	Itpkb	Ramp3
		2310058D17Rik	Ddit4	Izumo4	Rassf1
		2410022L05Rik	Ddt	Jdp2	Rassf4
		2610002J02Rik	Defb1	Jub	Rdh5
		2610100L16Rik	Degs1	Klf9	Retn
		2700078E11Rik	Dhrs13	Klhl21	Rhov
		2900026A02Rik	Dnaja1	Klra2	Rhox2c
		3200002M19Rik	Dnaja4	Lama3	Rlf
		4930405J17Rik	Dnajb1	Lamc3	Rnf145

		4930563M20Rik	Dpep1	Larp1b	Rnf183
		5730507C01Rik	Dpp7	Lbr	Ropn1l
		5730577I03Rik	Dqx1	Letm1	Rpp40
		A730020M07Rik	Dusp1	LOC100039753	Scd2
		AB099516	Eid1	LOC100861683	Senp5
		Abhd14a	Entpd2	LOC100862607	Serinc2
		Abi3bp	Entpd7	LOC100862646	Serpina1c
		Acaca	Ethe1	LOC666692	Sim2
		Acad9	Evc	Lyrm2	Slc10a6
		Acat1	Exoc5	Lztr1	Slc1a4
		Acsm2	Ext1	Map2k6	Slc25a38
		Afmid	F13b	Map3k6	Slc26a4
		Ahsa2	Fa2h	Mapk13	Slitrk6
		Aldh3a2	Fam100a	Mcl1	Sly
		Alk	Fam103a1	Med18	Smoc2
		Amdhd2	Fam107a	Mettl7a3	Smpdl3a
		Anapc16	Fam176a	Mir1186	Snap29
		Ankrd44	Fam187b	Mir145	Snhg11
		Aoah	Fbxo36	Mir1907	Snord70
		Arhgef10l	Fhit	Mir1948	Snord90
		Arpc5l	Fkbpl	Mir200b	Sp9
		Asb13	Fpr-rs4	Mir24-1	Spock3
		Ass1	Frmd4b	Mir26b	Srd5a2
		Atp6v0d2	Frzb	Mir532	Srebf1
		B230216N24Rik	Fuca2	Mknk2	St3gal5
		B4galt6	Fxyd4	Mmab	Stbd1
		Bcat1	Gadd45g	Mrpl14	Stk30
		Bcat2	Gbp6	Mug2	Ston1
		Bst1	Gcat	Mvd	Stxbp5l
		C4a	Gjb2	Myh10	Synpr
		Cables1	Gm10088	Mylk	Tchhl1
		Cacybp	Gm10579	Nat8	Tesk2
		Car3	Gm10914	Nceh1	Tmco3
		Ccdc6	Gm11362	Nphp1	Tmed10
		Ccdc85c	Gm13139	Npnt	Tmem207
		Cct8l1	Gm13251	Nr1d1	Tmem28
		Cdh13	Gm15348	Nt5c3	Tnfsf15
		Cdkn2aip	Gm17771	Nudt8	Tprn
		Ces1e	Gm19325	Obfc2a	Trappc9
		Chordc1	Gm19327	Obox2	Trav12-2
		Chrdl1	Gm19958	Olfr1282	Trav9d-3
		Chrna4	Gm3579	Olfr706	Trp53inp1
		Chst11	Gm4956	Olfr724	Trpv4
		Chst7	Gm5069	Olfr733	Tsga10
		Cib1	Gm5168	Olfr825	Ttll3
		Ckb	Gm5784	Olfr849	Ttll7
		Cldn24	Gm6976	Papss1	Uap1l1
		Cldn7	Gm6996	Pcdhb11	Ubb
		Clec2h	Gm7092	Pcsk6	Vmn1r198
		Cml2	Gm9805	Pdk3	Vmn1r20
		Cmtm6	Gpr133	Penk	Vmn1r42
		Cntnap2	Gpr75	Pfn3	Vmn2r41
		Cntnap5a	Gramd1b	Pgm5	Vmn2r61

		Col6a5	Gstz1	Pgp	Vmn2r67
		Cpxm2	H47	Pi15	Wasf3
		Crot	Herc4	Pim3	Wdr55
		Csgalnact1	Hhatl	Pknox1	Wrb
		Ctla2b	Hist1h2bn	Plcxd2	Xpnpep2
		Ctxn3	Hist2h3c2	Plekha6	Zbtb16
		Cxcl14	Hmgb1-rs17	Pomp	Zfp317
		Cyb561	Hmgcr	Ppic	Zfp442
		Cyp2d12	Hmox1	Ppp1r16b	Zfp600
		Cyp2f2	Hrg	Ppp1r1b	Zfp937
		Cyp4f14	Hyi	Ptger3	Zhx3
		Cyp51	Igfbp6	Pyroxd2	Zmynd10
**CKOxHKO**	**277**	1700007J10Rik	Cphx	Gm5635	Olfr419
		2010001M06Rik	Crb1	Gm5796	Olfr427
		2010109A12Rik	Creb3l1	Gm6445	Olfr61
		2310042E22Rik	Cts3	Gm7056	Olfr705
		2610028E06Rik	Cts8-ps	Gm7358	Olfr768
		3010003L21Rik	Cyp2a22	Gm9199	Pank3
		3010026O09Rik	Cyp2a5	Gm949	Pbld2
		3110045C21Rik	Cyp2c67	Gm9961	Pcsk9
		4732419C18Rik	Cyp2c69	Gpam	Piwil2
		4921515J06Rik	Cyp2d9	Gpr155	Pkd1l2
		4922505E12Rik	D14Ertd449e	Grid2ip	Plac1
		4930430J02Rik	D330028D13Rik	Grm6	Pllp
		4930433N12Rik	Dcpp2	Grpr	Pnck
		4930463O16Rik	Defa24	H1fx	Ppp2r5b
		4930483J18Rik	Defa-rs1	Hif3a	Pqlc2
		4930528G23Rik	Dera	Hist1h3h	Prh1
		4933408J17Rik	Dgkb	Hook2	Prkd3
		4933421A08Rik	Dlk2	Ide	Prss21
		4933430I17Rik	Doc2b	Ifna6	Ptgds
		4933433C11Rik	Dscaml1	Ifnk	Ptprq
		5730528L13Rik	Dusp14	Ifnz	Rab11b
		5930412G12Rik	Dusp23	Il22ra1	Rab33a
		6030468B19Rik	E130215H24Rik	Il5ra	Rasd1
		6330415B21Rik	Ear5	Ins2	Rasl10b
		7530416G11Rik	Ell3	Isl2	Rd3
		9130209A04Rik	Elovl2	Kif2b	Reln
		A2m	Epo	Kis2	Rps2
		A830010M20Rik	Eps8l3	Lce1h	Scand1
		Abca14	Erbb3	Lefty1	Scrn1
		Abhd3	F12	Lhx2	Scrn2
		Acmsd	Fam13a	LOC100047957	Sctr
		Adam23	Fam13c	LOC100861572	Selm
		Adap1	Fbp2	LOC100861893	Sftpa1
		Adcy2	Fbxo46	LOC433461	Siglece
		Agmat	Fndc5	LOC626049	Slc16a10
		Aldh1l2	Fnip2	LOC670059	Slc17a4
		Aldh4a1	Foxq1	Lrrc50	Slc18a1
		Angptl4	G0s2	Lypd3	Slc33a1
		Apoh	Gabrb3	Masp1	Slc6a5
		Apold1	Gatsl2	Meig1	Snca
		Arsg	Gip	Mir1192	Sncb

		Arx<br>Azi1<br>BC048602<br>BC051070<br>BC051077<br>BC067074<br>Bean1<br>Bid<br>Bms1<br>Bnipl<br>C130050O18Rik<br>C330046G03Rik<br>C530044C16Rik<br>Caly<br>Camk1g<br>Car9<br>Cbr2<br>Ccbl1<br>Ccdc164<br>Cckbr<br>Ccl26<br>Ces2h<br>Clip4<br>Cntn5<br>Col6a6<br>Col8a1<br>Colec10<br>Comt<br>Coro1c	Gm10318<br>Gm10509<br>Gm11564<br>Gm12248<br>Gm14407<br>Gm15070<br>Gm15315<br>Gm15645<br>Gm15983<br>Gm16157<br>Gm16677<br>Gm17416<br>Gm19277<br>Gm19281<br>Gm19462<br>Gm1976<br>Gm2046<br>Gm22<br>Gm2837<br>Gm2921<br>Gm3086<br>Gm3238<br>Gm5099<br>Gm514<br>Gm5329<br>Gm5346<br>Gm5475<br>Gm5480<br>Gm5591	Mir124a-2<br>Mir133a-2<br>Mir1945<br>Mir212<br>Mir3091<br>Mir486<br>Mir5109<br>Mir598<br>Mir717<br>Mir9-1<br>Mir92b<br>Mpped2<br>Mtmr7<br>Myog<br>Naa11<br>Narf<br>Ncrna00086<br>Neurod1<br>Nod2<br>Npm3<br>Nt5dc2<br>Ntn1<br>Olfm3<br>Olfr1061<br>Olfr1131<br>Olfr1219<br>Olfr1301<br>Olfr315<br>Olfr398	Snx20<br>Spats2l<br>Spink8<br>Sult1b1<br>Susd4<br>Syna<br>Sys1<br>Tbx10<br>Timm8a1<br>Tmem169<br>Tmem25<br>Tmem64<br>Tmigd1<br>Tram2<br>Trh<br>Tstd2<br>Tsx<br>Ttc39a<br>Ttyh2<br>Ucn2<br>Ugt3a1<br>Vmn1r16<br>Vmn1r82<br>Wnt2b<br>Zfp239<br>Zmynd12
