# Supplementary material for: Loss of Renal Tubular PGC-1α Exacerbates Diet-Induced Renal Steatosis and Age-Related Urinary Sodium Excretion in Mice
Source: PLoS One. 2016 Jul 27;11(7):e0158716. doi: 10.1371/journal.pone.0158716 (PMC4963111; doi:10.1371/journal.pone.0158716)
Supplement: S3 Table — List of genes significantly up-regulated (p<0.05, FC<1.2) between chow-fed control (CWT), chow-fed NiPKO (CKO), HFD-fed control (HWT) and HFD-fed NiPKO (HKO) groups. The heading “Groups” lists in which comparison the relevant gene(s) are found to be up-regulated. (PDF) [file pone.0158716.s009.pdf]

S3 Table

Labels	Number of genes	Genes			
**CWTxCKO**	**2**	Bex4			
**CWTxHWT**		Cpn1			
**HWTxHKO**					
**CKOxHKO**	**2**	Mogat2			
**CWTxCKO**		Trf			
**HWTxHKO**					
**CKOxHKO**	**2**	Agxt2l1			
**CWTxCKO**		Mgst1			
**CWTxHWT**					
**CKOxHKO**	**5**	Ccrn4l			
**CWTxHWT**		Cd19			
**HWTxHKO**		Cdca7l			
		Hp			
		Serpina3n			
**CWTxCKO**	**82**	5330417C22Rik	Clu	Hkdc1	Psat1
**HWTxHKO**		Abca13	Cyp2d12	Hpse2	Ptpn22
		Abcd4	D10Bwg1379e	Il17rb	Rab42-ps
		Abp1	D630023F18Rik	Itgb4	Rgs11
		Acot11	Dapp1	Kif6	Rhou
		Acy1	Dgkb	Lgals3bp	Sectm1b
		Aebp1	Emb	Limd2	Slc8a1
		Aldh1l2	Eps8l1	LOC621549	Snhg11
		Angptl7	Etnk2	Lxn	Snx31
		Arl4a	Fads1	Mboat1	Spink8
		Arnt2	Fetub	Mt2	Stap1
		Atp6v1g3	Foxred2	Muc1	Stk32b
		BC064078	Gabrb3	Myo3b	Sult1c2
		Blnk	Gamt	Myof	Susd1
		C2	Gm1332	Ngfrap1	Susd2
		C8a	Gm4952	Nlrc4	Tmem97
		Car3	Gpc3	Npl	Trpc5
		Casr	Gpr30	Nup93	Ttr
		Ccdc3	Gulo	Olfr1286	Zcchc16
		Cdhr2	Hexa	Pamr1	
		Ces1g	Hist2h2be	Papln	
**CWTxCKO**	**85**	1700003F12Rik	Col9a3	Krt34	Pla2g10
**CWTxHWT**		1700029H14Rik	Cxcl1	Lce3c	Pou2af1
		2810025M15Rik	Dkk4	Lcn6	Prph2
		3300005D01Rik	Drd5	LOC100861931	Prss35
		4930444G20Rik	Efemp1	Lzts1	Rasd2
		4930447K03Rik	Gli1	Maged2	Rbp4
		4930591A17Rik	Gm11564	Mir184	Sema3c
		4933433G19Rik	Gm13271	Mir486	Serpina3h
		5830473C10Rik	Gm13430	Mir551b	Slc24a4
		7530416G11Rik	Gm16197	Mir92b	Slc39a5
		A430107O13Rik	Gm16519	Mybpc2	Sox10
		Adam24	Gm5154	Myod1	Sprr2e
		Adamts13	Gm7102	Olfr1226	Svs5

		Adcy2	Gpr31b	Olfr1428	Syt5
		Angpt4	Grin3b	Olfr1502	Tcf24
		Arhgap33	Gsdmcl2	Olfr382	Tex19.2
		B3gnt8	Gsx1	Olfr410	Tmcc2
		BB319198	Igsf10	Olfr93	Trip13
		Bpifb2	Kcna2	Orm1	Trpd52l3
		Cd300lh	Kcnn3	P2rx2	Ugt2b34
		Cdh24	Klhdc1	Pcdh8	Zp4-ps
		Cldn11			
**CWTxHWT HWTxHKO**	**1**	Lrrc61			
**CKOxHKO HWTxHKO**	**61**	A730028G07Rik	Fkbp1b	Klk1b8	Rtn1
		Aacs	Foxq1	Maob	Slc16a14
		Adamts16	Gbp7	Mir10a	Slc4a9
		Arl13b	Gm13315	Mir3101	Snora20
		Arsj	Gm20236	Mir532	Socs2
		AU041133	Gm20257	Mrpl27	Sp4
		C030013D06Rik	Gm3219	Ntn4	Tcp1
		C9	Gm7429	Ogdhl	Tet2
		Cbfb	Gpr64	Pappa	Try5
		Ccdc21	Hoxb3	Pik3r3	Tspan6
		Ccl9	Ikzf4	Pilrb1	Vsig1
		Ces2g	Il7r	Prr5	Zfp180
		Cidec	Irgm2	Rasgrp1	Zfp449
		Defb2	Jarid2	Riok1	Zfp52
		Dleu2	Kif23	Rpl5	Zwilch
		Fcamr			
**CKOxHKO CWTxHWT**	**99**	4930572J05Rik	Dzip1	Klk1b7-ps	Slc25a25
		4933409K07Rik	Eci1	Leprel1	Slc2a5
		9530091C08Rik	Ednra	Med24	Slc5a10
		Abcb1a	Egln3	Mmp12	Slc6a4
		Abcb1b	Eid3	Mpzl2	Slc7a12
		Acnat1	Fabp5	Mybl1	Smad7
		Agt	Fam78a	Nbeal2	Snora16a
		Aim1l	Fgf18	Nmrk1	Snora17
		Apob	Gc	Nrxn2	Snora31
		Apoe	Gldc	Pbx4	Snora70
		Atr	Gm13298	Pck1	Spns2
		Brca2	Gm20342	Pdk1	St3gal1
		C3	Gm6614	Pdxk	Taf1d
		C730036E19Rik	Gm7457	Pklr	Tfcp2l1
		Car14	Gria3	Pla2g5	Tmie
		Ccdc141	Gsto1	Prlr	Tnxb
		Cdc14a	Hmgcs2	Ptgs2	Trim34a
		Cdkl5	Hoxa1	Pvalb	Tspan18
		Cklf	Hoxc8	Rdh18-ps	Ugt1a10
		Cml1	Id1	Rgs5	Vkorc1
		Col27a1	Id2	Serinc5	Wee1
		Cp	Id3	Slc13a2	Wsb1
		Dak	Immp2l	Slc13a3	Zfp161
		Dhrs7	Kcna4	Slc16a6	Zfp454
		Dusp9	Kcnip2	Slc19a3	

**CWTxCKO**	**338**	1500011B03Rik	Ctsz	Has3	Olfr437
		1700007J10Rik	Cxcl13	Heph	Olfr525
		1700025N23Rik	Cxcl16	Hhip	Olfr541
		1700121N20Rik	Cyp26c1	Htr3a	Olfr66
		1810046K07Rik	Cyp2a12	Hvcn1	Olfr661
		2010002N04Rik	Cyp2c69	I830127L07Rik	Panx1
		2300005B03Rik	Cyp2d11	Ifna7	Pbld2
		2310007B03Rik	Cyp2r1	Igf2bp1	Pcdh17
		2610016A17Rik	D17H6S56E-5	Ighv1-72	Pdcd1
		3110035E14Rik	D330028D13Rik	Igh-VJ558	Pdilt
		3110082I17Rik	Dact3	Iglv1	Pdlim3
		3930402G23Rik	Defa-rs1	Il17c	Pgrmc1
		4922505E12Rik	Defb35	Il5ra	Pkd1l2
		4930433N12Rik	Defb42	Irx6	Plagl1
		4930459I23Rik	Dgkz	Isl2	Plin4
		4930474N09Rik	Dnahc2	Itgam	Pmaip1
		4930483J18Rik	Dnajc5b	Itgb2	Ppargc1a
		4930589P08Rik	Dnali1	Kcne1l	Pqlc2
		4931403M11Rik	Dscaml1	Kcnh3	Prh1
		4933405L10Rik	Dusp2	Kcnh7	Prom1
		4933408J17Rik	E030030I06Rik	Kcnk13	Psg19
		4933429O19Rik	E230025N22Rik	Kcnk7	Psors1c2
		4933430I17Rik	Ear5	Kif18b	Pspn
		4933433C11Rik	Efna3	Klrb1f	Ptgds
		5430437J10Rik	Eps8l3	Krt24	Pycr1
		9030619P08Rik	Fam13c	Krt31	Rab9b
		9130008F23Rik	Fam171a2	Lce1i	Rasgef1a
		9230117E06Rik	Fam55c	Lcn8	Rd3
		9530026F06Rik	Fbll1	Lefty1	Reln
		A530006G24Rik	Fcgbp	Leprel2	Rhobtb3
		A530020G20Rik	Fgf22	Lhfpl2	Rhox3g-ps
		A630038E17Rik	Fgfbp1	Lhfpl4	Rnf39
		Abpa	Fkbp6	LOC100040974	Rpl13
		Acat3	Foxi1	LOC100861572	Scrn1
		Ace2	Foxi3	Lpar2	Sctr
		Actn3	Fxc1	Lrfn1	Sdk1
		Adam23	G630093K05Rik	Lrrc50	Serp2
		Adprhl2	Galnt13	Lrrn3	Serpinb9
		Adra2c	Galnt9	Lrrtm1	Serpind1
		Afp	Gcsh	Ltbp2	Sez6l2
		Ano9	Gdf2	Luzp2	Sfn
		Apcdd1	Gjb4	Lypd3	Sftpb
		Apom	Gjc1	Mab21l3	Shd
		Arhgap23	Gltpd2	Mageb16-ps1	Sidt1
		Arhgdig	Gm10100	Magi2	Six6
		Asb11	Gm10318	Man2b2	Slc17a4
		AU016765	Gm10584	March10	Slc17a9
		B4galt7	Gm10636	Med7	Slc34a2
		Bach2	Gm10782	Mir124a-2	Slc6a5
		Barx2	Gm1096	Mir187	Slc7a15
		BC067074	Gm11541	Mir191	Snca
		Bco2	Gm12248	Mir194-1	Sncb
		Bdnf	Gm13080	Mir194-2	Sostdc1

		Bmp15	Gm13286	Mir1943	Sox9
		Bms1	Gm14047	Mir3091	Sp6
		Bpifb9b	Gm14407	Mir337	Sssca1
		Btnl1	Gm17540	Mir421	Stab2
		C130050O18Rik	Gm19462	Mir450-2	Syna
		C1s	Gm1976	Mir467e	Tas1r2
		C230036H16Rik	Gm3086	Mir574	Tbx22
		C330018A13Rik	Gm3279	Mir669m-1	Tdpoz2
		C630004H02Rik	Gm4371	Mir717	Tgfa
		C86187	Gm4651	Mob3a	Tgm5
		C86695	Gm4871	Mreg	Tm4sf19
		Capn8	Gm4934	Msx2	Tm7sf3
		Catsperg1	Gm5094	Mt1	Tmem169
		Cbr2	Gm5099	Muc2	Tmem235
		Ccdc121	Gm5334	Myh6	Tmem8c
		Ccdc164	Gm5591	Myo5a	Tnni2
		Ccl26	Gm6300	Naa11	Trdn
		Cd300a	Gm6978	Ncan	Trim45
		Cdsn	Gm7168	Ncrna00086	Trim59
		Cerkl	Gm7361	Neu4	Trpc7
		Ces2h	Gm8439	Nkx2-9	Trpm5
		Cit	Gm9782	Notum	Tshr
		Col3a1	Gng4	Npm3	Tsx
		Col5a2	Gpr50	Nqo1	Vmn1r4
		Cpz	Grem1	Ntn3	Vmn2r3
		Crabp1	Grin1	Nusap1	Wbscr28
		Crb1	Grin2c	O3far1	Wdr89
		Creb3l1	Grm6	Olfr1219	Wnt4
		Cryaa	Gstm3	Olfr1301	Zfp239
		Crybb3	Gucy1a3	Olfr1487	Zswim3
		Ctps	H1fx	Olfr294	
		Ctsc	H2afb1	Olfr331	
**HWTxHKO**	**263**	1190003J15Rik	D630024D03Rik	Igkv4-72	Samd5
		1700029I01Rik	D930048N14Rik	Impact	Sc4mol
		1700123O21Rik	Dcaf12l1	Insrr	Scd2
		1700125D06Rik	Defb29	Itih2	Scel
		1810063I02Rik	Dnahc5	Izumo4	Sectm1a
		2010001M09Rik	Dnajb2	Jdp2	Senp5
		2310081J21Rik	Dsp	Kcnj13	Serpina1c
		2810474O19Rik	Dtna	Kcnk5	Serpina1d
		4930502E18Rik	Dusp1	Klra2	Serpinc1
		5330426P16Rik	E030024N20Rik	Krt14	Sim1
		5730577I03Rik	E230016K23Rik	L1cam	Slc1a3
		9030025P20Rik	Eid2	Lama1	Slc1a4
		9230110C19Rik	Elfn1	Lama3	Slc26a4
		AB099516	Endou	Lamc3	Slc38a6
		Ablim3	Ephb2	Lass4	Slco3a1
		Ace	Esrp1	Ldhd	Sned1
		Actn1	F13b	Lipg	Snord104
		Adam11	Fabp7	LOC621118	Snord70
		Adamtsl3	Fam103a1	Lrp11	Sntb1
		Agxt2	Fam107a	Maoa	Soat1
		Akr1c13	Fam174b	Mgat4a	Sorbs2

		Amdhd2	Fasn	Mgmt	Spcs2
		Amn	Fbxo36	Mir1186	Sprr2a1
		Ank2	Fbxo6	Mir145	Sqle
		Anxa9	Fbxw9	Mir200b	Srgap3
		Aoah	Fdps	Mir24-1	St6gal1
		Asah1	Fuca2	Mir669j	Sytl2
		Atp6v0e2	Fxyd3	Mirlet7c-2	Sytl5
		Atxn7l1	Fxyd4	Mknk2	Tagap
		Atxn7l2	Galns	Myef2	Tagln2
		Avpr1a	Gcat	Mylk	Tceal1
		B4galt1	Gdf11	Nat2	Tchhl1
		Bambi-ps1	Glb1l2	Nhlrc4	Terc
		Bcat1	Gm10416	Nipal2	Tfap2b
		Bmpr1b	Gm11559	Npc2	Tmed6
		Btg2	Gm12824	Npr2	Tmem207
		C130026I21Rik	Gm13139	Nrbp2	Tmem30b
		C1qtnf3	Gm13251	Nrg1	Tox3
		C330002G04Rik	Gm15348	Olfr1337	Trav9d-3
		Camk1d	Gm20115	Olfr694	Trmt112
		Capn6	Gm2423	Olfr810	Trp53inp1
		Car13	Gm5432	Parp11	Tspan13
		Cav1	Gpr110	Parp8	Tspan8
		Cbs	Gpr112	Pgm5	Tstd1
		Ccdc159	Gpr39	Phf15	Tuba1a
		Cckar	Gprasp2	Phgr1	Uap1l1
		Cdc25b	Gpt	Phlda2	Ugt2a3
		Cdc42se1	Grhl2	Pisd-ps2	Vgll4
		Cdk14	Gstp1	Pkib	Vill
		Cdkn1a	H2afj	Plcg2	Vmn1r27
		Ceacam2	H2-DMb2	Plcxd2	Vmn1r32
		Chrdl1	Hbegf	Plxnb1	Vmn1r45
		Chrna4	Hebp1	Ppic	Wbp5
		Chst11	Hepacam2	Ppp1r16b	Wnk4
		Cldn8	Hexb	Ppp1r1a	Xbp1
		Cldn9	Hist1h2bn	Psd4	Zbtb16
		Ctxn1	Hist1h4i	Ptpn13	Zc4h2
		Cxcl3	Hist2h3b	Pvr	Zfp335
		Cxx1c	Hist2h3c2	Pzp	Zfp442
		Cyp2a4	Hmox1	Rab32	Zfp46
		Cyp2d40	Hmx2	Rcan1	Zfp760
		Cyp4f16	Hoxb8	Rgn	Zfp781
		Cyp4v3	Hyi	Rgs2	Zfp810
		Cyp7b1	Ighg	Rhox2c	Zfp873
		Cys1	Igk-V1	Rnf145	Zfp937
		D430041D05Rik	Igkv4-59	Ropn1l	
**CWTxHWT**	**326**	1700001G17Rik	Cox6b2	Gm6086	Pla2g16
		1700041M05Rik	Creb3l3	Gm6222	Pldi
		1700067G17Rik	Cstf2	Gm7385	Ppcs
		1700095J03Rik	Cygb	Gm9442	Rab27b
		1700110C19Rik	Cyp2d13	Gm9979	Rab30
		2010300F17Rik	Cyp2d26	Gpr149	Rab40b
		2300002M23Rik	Cyp4a10	Gpr27	Ralgapa2
		2410016O06Rik	Cyp4a14	Gprin3	Rasa4

		2610018G03Rik<br>2610020C07Rik<br>2810405K02Rik<br>2810408M09Rik<br>3230401D17Rik<br>3830408C21Rik<br>4632404H12Rik<br>4930401B11Rik<br>4930467K11Rik<br>4930524N10Rik<br>4930540M05Rik<br>4930563F08Rik<br>4930583I09Rik<br>4931403G20Rik<br>4932416K20Rik<br>4933411K16Rik<br>4933422A05Rik<br>5430421F17Rik<br>5730435O14Rik<br>5730457N03Rik<br>9430015G10Rik<br>9530026P05Rik<br>A430093F15Rik<br>A830060N17<br>Abcc4<br>Abcg1<br>Abhd15<br>Acaa1b<br>Acaa2<br>Acadvl<br>Acot2<br>Acot3<br>Acot4<br>Acot6<br>Acsf2<br>Agpat9<br>Aldh1a1<br>Aldh1a7<br>Alyref2<br>Apoa2<br>Apobr<br>Apoc3<br>Arhgap19<br>Arhgef9<br>Armcx4<br>Asrgl1<br>Atp12a<br>Atp2a1<br>Atp2a3<br>AU040972<br>AW011738<br>AY358078<br>BC066135	Cyp4a31<br>D0H4S114<br>D630003M21Rik<br>D630042P16Rik<br>D830015G02Rik<br>D930030D11Rik<br>Ddhd2<br>Dgkh<br>Dub1<br>Dyx1c1<br>E130112N10Rik<br>E130317F20Rik<br>Ear2<br>Ech1<br>Edil3<br>Efcab4b<br>Eif2s3y<br>Eif4a3<br>Emr4<br>Ern2<br>Esp3<br>Esr2<br>Evpl<br>Expi<br>Fabp1<br>Fam166b<br>Fam18a<br>Fam50b<br>Fam5b<br>Fam83g<br>Fbxl16<br>Fchsd1<br>Fgf14<br>Fgf16<br>Fgfrl1<br>Fndc8<br>Foxb2<br>Foxj2<br>Frmd5<br>Fsd1<br>Fus<br>Fut4-ps1<br>G6pd2<br>Gal3st2<br>Gan<br>Gapdhs<br>Gast<br>Gbe1<br>Gbx2<br>Glp1r<br>Gm10503<br>Gm10639<br>Gm10696	Grin2d<br>Grk4<br>Gsta1<br>Gsx2<br>Hbb-bh2<br>Hlf<br>Hnrnpd<br>Homer3<br>Hoxc12<br>Idi2<br>Ifi44<br>Igf2bp2<br>Il25<br>Jph1<br>Katna1<br>Kcnmb3<br>Khk<br>Kif15<br>Klhl34<br>Krt6b<br>Kynu<br>Large<br>Lcn2<br>Ldlrad2<br>Lgals4<br>Lhx8<br>LOC100861753<br>LOC664924<br>Lst1<br>Map3k9<br>Mcpt-ps1<br>Mfsd12<br>Mgll<br>Mif<br>Mir1190<br>Mir1930<br>Mir1938<br>Mir301b<br>Mir3070b<br>Mir3073<br>Mir3098<br>Mir339<br>Mir342<br>Mir455<br>Mir466g<br>Mir5125<br>Mir695<br>Mir744<br>Mirlet7a-2<br>Mpl<br>Ms4a1<br>Mup5<br>Ngfr	Rasal3<br>Ren1<br>Rgs9bp<br>Rhbdl3<br>Rhox4f<br>Ripk3<br>Rnu11<br>Rpa2<br>Rps13<br>Rps19<br>Rps8<br>Rrm1<br>Sh2d1b2<br>Sh3gl3<br>Slc12a5<br>Slc13a5<br>Slc22a20<br>Slc25a20<br>Slc25a22<br>Slc27a1<br>Slc5a1<br>Slfn2<br>Slitrk4<br>Slx1b<br>Smad9<br>Snora30<br>Snora41<br>Snord55<br>Snord88c<br>Sox30<br>Spata9<br>Spatc1<br>Spc25<br>Spib<br>Spint4<br>Spp2<br>Spred3<br>Sprr1b<br>Ssxa1<br>Stfa2l1<br>Syt3<br>Tcf19<br>Tet1<br>Tgm2<br>Tigd3<br>Timp1<br>Tm6sf2<br>Tmem59l<br>Tmem91<br>Tnfaip8l3<br>Trim12c<br>Trim62<br>Trpv2

		BC089597	Gm10787	Nr0b1	Tspan2
		Bcl11b	Gm10923	Olfr1015	Tubb5
		Bhlhe22	Gm10941	Olfr1320	Tubg2
		Btbd19	Gm11110	Olfr1335	Txndc8
		C2cd4a	Gm11744	Olfr156	Uchl1
		C5ar1	Gm12356	Olfr325	Ucp2
		Cc2d2b	Gm13369	Olfr401	Usp49
		Cd14	Gm14762	Olfr522	Vmn1r2
		Cd5	Gm15628	Olfr582	Vmn2r20
		Cd80	Gm15867	Olfr859	Vwce
		Cdkn1c	Gm19654	Otp	Wdhd1
		Cdt1	Gm20095	Padi1	Wisp1
		Cep78	Gm20279	Pax6os1	X99384
		Ces1d	Gm20382	Pcdhb7	Zfp423
		Chaf1b	Gm2564	Pcx	Zfp488
		Chchd2	Gm2863	Pdap1	Zfp612
		Chrm1	Gm3776	Pde5a	Zfp648
		Clec18a	Gm4301	Pex11a	Zfp712
		Clic6	Gm5237	Phf11	Zfp932
		Cnr1	Gm5451	Pif1	
		Coro1a	Gm5478	Pknox2	
**CKOxHKO**	**367**	1500012F01Rik	Entpd1	Maml3	Shroom1
		1520402A15Rik	Ep400	Map3k12	Skil
		1700020D05Rik	Ephb4	Mapk4	Skp2
		1700034F02Rik	Ercc8	Mapkapk3	Slc20a1
		1700034I23Rik	Ero1lb	March3	Slc22a15
		1700052K11Rik	Ezh2	Marcksl1-ps4	Slc22a8
		2210408F21Rik	Fabp4	Marveld1	Slc23a3
		2310003L22Rik	Fam164c	Mcm4	Slc25a14
		2310057M21Rik	Fam83b	Mdm1	Slc35f1
		2410004N09Rik	Fastkd1	Mest	Slc38a2
		2610017I09Rik	Fermt1	Mfsd7b	Slc45a3
		2610528B01Rik	Fga	Mgl2	Slc7a6
		2810408B13Rik	Flrt1	Mgp	Slco4a1
		4930405O22Rik	Frk	Mir181a-2	Smad5
		4930480K23Rik	Fsd1l	Mir188	Sncg
		4930522L14Rik	Fv1	Mir1927	Snora15
		5730409K12Rik	Fxr2	Mir1949	Snora21
		9130020K20Rik	Fzd7	Mir19a	Snora23
		9930111J21Rik2	Gas6	Mir1b	Snora2b
		A230072C01Rik	Gca	Mir218-1	Snora69
		A630050E04Rik	Gcet2	Mir3473b	Snora75
		A730098P11Rik	Glod5	Mir872	Snord11
		A930033H14Rik	Glul	Mis12	Snord12
		Abcd2	Gm10002	Mmd	Snord1b
		Abi3	Gm10589	Mndal	Snord35b
		Actg1	Gm13363	Mpzl3	Snord52
		Adamts1	Gm14057	Mrpl12	Snord61
		Adat1	Gm14420	Msr1	Snord68
		Adcy4	Gm15293	Mthfs	Snord87
		Adra2b	Gm15483	Mtm1	Snord89
		Akr1c20	Gm15535	Mustn1	Snx32
		Anapc11	Gm16523	Nampt	Snx33

		Ankra2	Gm17753	Ncrna00085	Sox18
		Aoc2	Gm19265	Neat1	Sp100
		Apbb1ip	Gm19537	Neurl1b	Spag5
		Aqp4	Gm19551	Nfkb1	Srl
		Arhgap25	Gm20004	Nfkb2	Srsf7
		Atf7	Gm20022	Nicn1	Stc1
		Atp5h	Gm20186	Nkap	Susd3
		Atp8a1	Gm20289	Nova1	Syne1
		Auts2	Gm3893	Nrp1	Tap2
		B230319C09Rik	Gm4567	Ntf3	Tceb1
		B230369F24Rik	Gm4759	Oas1a	Tek
		Bbs5	Gm6289	Olfm4	Tle4
		BC002059	Gm8842	Olfr1336	Tmco4
		BC027231	Gm8995	Olfr776	Tmem108
		BC037704	Gm9958	Ophn1	Tmem156
		BC065397	Gna14	Osmr	Tmem171
		Bhlhe40	Gpcpd1	P2ry14	Tmem173
		Birc2	Gpnmb	Pag1	Tmem200b
		C030046E11Rik	Gprc5b	Paqr3	Tnip2
		C630016N16Rik	Hadhb	Pcdhga11	Trim30a
		Cadps2	Heg1	Pcgf5	Trim32
		Ccl2	Hells	Peg13	Trim6
		Ccl3	Hes1	Per2	Trip10
		Ccnd1	Hist1h4k	Piga	Try10
		Ccnd2	Hivep2	Plekhg5	Tshz3
		Ccnt1	Hnf1b	Plekho1	Tsku
		Ccrl2	Hoxb4	Plin1	Tspan1
		Cd180	Hoxb5	Pln	Tspan4
		Cd22	Hoxc9	Pltp	Tyms
		Cd79a	Hoxd9	Pon3	Ugt2b37
		Cd8b1	Hyal1	Ppan	Ugt2b5
		Cdc25a	Icam1	Ppp2r2a	Uhrf1bp1
		Cdk17	Ifi27l1	Pprc1	Unc13c
		Ceacam14	Ikzf2	Praf2	Usp31
		Cebpa	Itga3	Prdm9	Usp40
		Cep170	Itga5	Prim1	Vcam1
		Chml	Itgax	Ptch1	Vmn1r71
		Chst1	Jun	Ptgs1	Wdr36
		Chst2	Kcnj2	Ptp4a1	Wdr76
		Clec14a	Kcnk1	Ptpla	Wwc1
		Clec2d	Kcnq1	Purg	Xdh
		Cox7b	Kctd12b	Pvrl1	Zbtb2
		Cpeb1	Klhdc7a	Rab6b	Zbtb38
		Crip1	Klhdc8a	Rab8b	Zdhhc2
		Crip2	Klkb1	Rad1	Zdhhc20
		Cwh43	Lad1	Rad9b	Zfp128
		Cxcl10	Larp4b	Rapgef3	Zfp229
		D5Ertd579e	Layn	Rasa3	Zfp429
		D730048I06Rik	Lep	Rasl11b	Zfp451
		D930015E06Rik	LOC100503141	Rel	Zfp503
		Dcaf12	LOC100505027	Rfc4	Zfp566
		Derl3	LOC100861803	Rnu73b	Zfp597
		Dhrs3	LOC100861921	Rny1	Zfp605

		Disp1<br>Dnalc1<br>Dnase1l3<br>Dpy19l1<br>Dsg2<br>Dusp11<br>E330009J07Rik	LOC100862007<br>LOC545261<br>LOC628147<br>Lpcat1<br>Lrig1<br>Mafb<br>Magohb	Rps17<br>Rsl1<br>Rtkn<br>Scarna3b<br>Scn4b<br>Scube3<br>Shmt1	Zfp667<br>Zfp788<br>Zfp879<br>Zranb3<br>Zscan2<br>Zxdb
