# Supplementary material for: Loss of Renal Tubular PGC-1α Exacerbates Diet-Induced Renal Steatosis and Age-Related Urinary Sodium Excretion in Mice
Source: PLoS One. 2016 Jul 27;11(7):e0158716. doi: 10.1371/journal.pone.0158716 (PMC4963111; doi:10.1371/journal.pone.0158716)
Supplement: S4 Table — Genes associated with KEGG pathways in CTRL vs. NiPKO animals. (PDF) [file pone.0158716.s010.pdf]

S4 Table

Chow - CTRL vs. NiPKO – Down-regulated genes					
**#term**	**Term name**	**p-value**	**Adjusted p-value**	**-log(p)**	**Positive ids**
mmu05012	Parkinson's disease (PD)	8.75E-36	1.65E-33	**32.78**	Cox7a1,ND6,LOC100046079,Atp5h,Atp5e,Uqcrfs1,Cycs,Ndufc1,Atp5g3,Cox5a,Ndufs1,Cox4i1,Uqcrh,Ndufa8,Ndufa4,Uqcrc1,Cyc1,Ndufv2,BC002163,Ndufs6,Sdhd,Cox7a2,Ndufa1,Ndufb8,Ndufa9,Uqcrb,Ndufs8,Cox7b,Uqcrq,Atp5d,Vdac2,Slc25a4,Ndufa6,Ndufs3,Atp5f1,Vdac3,Ndufb2,Sdhc,Ndufb5,Sdha,Ndufs2
mmu00190	Oxidative phosphorylation	2.39E-35	2.26E-33	**32.65**	Cox7a1,Cox10,ND6,LOC100046079,Atp5h,Atp5e,Uqcrfs1,Ndufc1,Atp5g3,Cox5a,Ndufs1,Cox4i1,Uqcrh,Ndufa8,Ndufa4,Atp5l,Uqcrc1,Cyc1,Atp4a,Ndufv2,BC002163,Ndufs6,Sdhd,Cox7a2,Ndufa1,Ndufb8,Ndufa9,Uqcrb,Ndufs8,Cox7b,Uqcrq,Atp5d,Ndufa6,Ndufs3,Atp5f1,Ndufb2,Sdhc,Ndufb5,Sdha,Ndufs2
mmu05016	Huntington disease (HD)	4.71E-31	2.97E-29	**28.53**	Cox7a1,LOC100046079,Atp5h,Atp5e,Uqcrfs1,Cycs,Ndufc1,Atp5g3,Cox5a,Ndufs1,Cox4i1,Uqcrh,Ndufa8,Ndufa4,Uqcrc1,Cyc1,Ndufv2,BC002163,Ndufs6,Sdhd,Cox7a2,Ndufa1,Ndufb8,Ndufa9,Uqcrb,Ndufs8,Cox7b,Uqcrq,Atp5d,Vdac2,Slc25a4,Ndufa6,Ndufs3,Atp5f1,Vdac3,Ndufb2,Sdhc,Polr2h,Ndufb5,Sdha,Ndufs2
mmu05010	Alzheimer's disease (AD)	2.11E-26	9.97E-25	**24.00**	Cox7a1,LOC100046079,Atp5h,Atp5e,Uqcrfs1,Cycs,Ndufc1,Atp5g3,Cox5a,Ndufs1,Cox4i1,Uqcrh,Ndufa8,Ndufa4,Uqcrc1,Cyc1,Ndufv2,BC002163,Ndufs6,Sdhd,Cox7a2,Ndufa1,Ndufb8,Ndufa9,Uqcrb,Ndufs8,Cox7b,Uqcrq,Atp5d,Ndufa6,Ndufs3,Atp5f1,Ndufb2,Sdhc,Ndufb5,Sdha,Ndufs2
mmu00020	The citrate cycle	9.63E-15	3.64E-13	**12.44**	Idh3a,Pdha1,Dlst,Dlat,Sdhd

	(TCA cycle, Krebs cycle)				,Pdhb,Cs,Fh1,Mdh2,Dld,Sdhc,Sdha,Idh3b
mmu04260	Cardiac muscle contraction	6.99E-08	2.2E-06	**5.66**	Cox7a1,LOC100046079,Uqcrfs1,Cox5a,Cox4i1,Uqcrh,Uqcrc1,Cyc1,Cox7a2,Uqcrb,Cox7b,Uqcrq
mmu00010	Glycolysis	8.17E-08	2.21E-06	**5.66**	Pfkm,Pfkp,Pgam1,Ldhb,Pdha1,Pgam2,Dlat,Pgk1,Hk1,Pdhb,Dld
mmu00620	Pyruvate metabolism	1.50E-05	0.000354	**3.45**	Me3,Ldhb,Pdha1,Dlat,Pdhb,Mdh2,Dld
mmu00250	Alanine, aspartate and glutamate metabolism	3.02E-04	0.006348	**2.20**	Got1,Gpt2,Adssl1,Glul,Ddo

**Chow - CTRL vs. NiPKO – Up-regulated genes**					
**#term**	**Term name**	**p-value**	**Adjusted p-value**	**-log(p)**	**Positive ids**
mmu00511	Other glycan degradation	0.00002377	0.004493	2.35	Hexa, Sox10, Foxi1, Neu4, Man2b2

**HFD - CTRL vs. NiPKO – Down-regulated genes**					
**#term**	**Term name**	**p-value**	**Adjusted p-value**	**-log(p)**	**Positive ids**
mmu00190	Oxidative phosphorylation	2.19E-20	4.1363E-18	**17.38**	Ndufa9,Cox7a1,Atp5h,Uqcrc1,Cox5a,Cox7a2,Ndufa4,Ndufs3,Ndufs8,Uqcrfs1,Atp5g3,Ndufa8,Cyc1,Uqcrh,Atp5k,Ndufc1,Cox10,Atp5f1,Uqcrq,LOC100046079,Atp5c1,Atp5e,Atp5g1,Ndufa1,Cox5b,Uqcrb
mmu05012	Parkinson's disease (PD)	7.45E-20	7.04477E-18	**17.15**	Ndufa9,Cox7a1,Atp5h,Uqcrc1,Cox5a,Cox7a2,Ndufa4,Ndufs3,Ndufs8,Uqcrfs1,Atp5g3,Ndufa8,Cyc1,Uqcrh,Ndufc1,Atp5f1,Uqcrq,LOC100046079,Atp5c1,Atp5e,Cycs,Slc25a4,Atp5g1,Ndufa1,Cox5b,Uqcrb
mmu05010	Alzheimer's disease (AD)	3.26E-18	1.80054E-16	**15.74**	Ndufa9,Cox7a1,Atp5h,Uqcrc1,Cox5a,Cox7a2,Ndufa4,Ndufs3,Ndufs8,Uqcrfs1,Atp5g3,Ndufa8,Cyc1,Uqcrh,Ndufc1,Atp5f1,Uqcrq,LOC100046079,Atp5c1,Atp5e,Cycs,Atp5g1,Ndufa1,Atp2a1,Cox5b,Gapdh,Uqcrb

mmu05016	Huntington disease (HD)	3.81E-18	1.80054E-16	**15.74**	Ndufa9,Cox7a1,Atp5h,Uqcrc1,Cox5a,Cox7a2,Ndufa4,Ndufs3,Ndufs8,Uqcrfs1,Atp5g3,Ndufa8,Cyc1,Uqcrh,Ndufc1,Atp5f1,Uqcrq,LOC100046079,Atp5c1,Atp5e,Cycs,Slc25a4,Atp5g1,Ndufa1,Cox5b,Uqcrb,Creb3l3
mmu04260	Cardiac muscle contraction	7.95E-08	3.00472E-06	**5.52**	Cox7a1,Uqcrc1,Cox5a,Cox7a2,Uqcrfs1,Cyc1,Uqcrh,Uqcrq,LOC100046079,Cox5b,Uqcrb
mmu00010	Glycolysis	1.16E-06	3.65533E-05	**4.44**	Tpi1,Ldhb,Pfkm,Dlat,Pgk1,G6pc,Pgam1,Pfkp,Gapdh
mmu00020	The citrate cycle	1.39E-04	0.00375555	**2.43**	Mdh2,Idh3a,Dlst,Fh1,Dlat
mmu00071	Fatty acid degradation	8.26E-04	0.0195103	**1.71**	Acaa2,Acsl3,Acadl,Acadvl,Hadha
mmu00030	The pentose phosphate pathway	1.21E-03	0.0254251	**1.59**	Pfkm,Pgls,Pfkp,G6pd2

**HFD - CTRL vs. NiPKO – Up-regulated genes**					
**#term**	**Term name**	**p-value**	**Adjusted p-value**	**-log(p)**	**Positive ids**
mmu05322	Systemic lupus erythematosus (SLE)	1.89E-05	0.00356441	**2.45**	C2,Hist1h2bn,Hist2h3b,Actn1,Hist2h3c2,Hist2h2be,C8a,Hist1h4i,C9,H2-DMb2,H2afj
mmu00260	Glycine, serine and threonine metabolism	2.10E-04	0.0198883	**1.70**	Gamt,Psat1,Cbs,Maoa,Maob
mmu00531	Glycosaminoglycan degradation	5.79E-04	0.036507	**1.44**	Hexb,Hexa,Hpse2,Galns
