# Supplementary material for: Loss of Renal Tubular PGC-1α Exacerbates Diet-Induced Renal Steatosis and Age-Related Urinary Sodium Excretion in Mice
Source: PLoS One. 2016 Jul 27;11(7):e0158716. doi: 10.1371/journal.pone.0158716 (PMC4963111; doi:10.1371/journal.pone.0158716)
Supplement: S5 Table — Genes associated with KEGG pathways CHOW vs. HFD treated animals. (PDF) [file pone.0158716.s011.pdf]

S5 Table

CTRL - CHOW vs. HFD – Down-regulated genes					
**#term**	**Term name**	**p-value**	**Adjusted p-value**	**-log(p)**	**Positive ids**
mmu00100	Steroid biosynthesis	1.79E-09	3.37758E-07	**6.47**	Sqle, Sc4mol, Dhcr24, Cyp51, Fdft1, Tm7sf2, Nsdhl, Dhcr7,
mmu00650	Butanoate metabolism	1.24E-08	1.17302E-06	**5.93**	Acsm3, Acsm1, Bdh1, Abat, Oxct1, Acsm2, Echs1, Aldh3a2, Acsm5, Acat1,
mmu00280	Valine, leucine and isoleucine degradation	1.24E-05	0.000782	**3.11**	Bcat1, Ivd, Abat, Oxct1, Bcat2, Echs1, Aldh3a2, Acat1,
mmu00900	Terpenoid backbone biosynthesis	1.74E-05	0.000822	**3.08**	Idi1, Fdps, Hmgcr, Mvd, Acat1,
mmu00380	Tryptophan metabolism	2.39E-04	0.009034	**2.04**	Aadat, Ido2, Echs1, Aldh3a2, Afmid, Acat1,
mmu00640	Propanoate metabolism	5.25E-04	0.016522	**1.78**	Abat, Acaca, Echs1, Aldh3a2, Acat1,
mmu00072	Synthesis and degradation of ketone bodies	1.85E-03	0.049988	**1.30**	Bdh1, Oxct1, Acat1,

CTRL - CHOW vs. HFD – Up-regulated genes					
**#term**	**Term name**	**p-value**	**Adjusted p-value**	**-log(p)**	**Positive ids**
mmu03320	PPAR signaling pathway	6.92E-09	1.30873E-06	**5.88**	Hmgcs2,Pck1,Sox10,Fabp5,Cyp4a10,Apoa2,Cyp4a31,Spib,Fabp1,Cyp4a14,Acaa1b,Apoc3,Slc27a1
mmu00071	Fatty acid degradation	5.50E-06	0.000519548	**3.28**	Acaa2,Sox10,Cyp4a10,Acadvl,Cyp4a31,Spib,Cyp4a14,Acaa1b
mmu00830	Retinol metabolism	9.82E-05	0.00618701	**2.21**	Ugt1a10,Cyp4a10,Aldh1a1,Cyp4a31,Spib,Ugt2b34,Cyp4a14,Aldh1a7
mmu00480	Glutathione metabolism	1.72E-04	0.0081363	**2.09**	Sox10,Mgst1,Gsto1,Spib,Rrm1,G6pd2,Gsta1
mmu04270	Vascular smooth muscle contraction	3.31E-04	0.00832382	**2.08**	Sox10,Cyp4a10,Adcy2,Pla2g10,Cyp4a31,Spib,Ednra,Agt,Pla2g5,Cyp4a14
mmu00860	Porphyrin and chlorophyll metabolism	4.34E-04	0.00832382	**2.08**	Sox10,Ugt1a10,Spib,Ugt2b34,Cp
mmu00590	Arachidonic acid metabolism	4.30E-04	0.00832382	**2.08**	Sox10,Cyp4a10,Pla2g10,Cyp4a31,Spib,Pla2g5,Cyp4a14,Ptgs2
mmu02010	ABC transporters	4.40E-04	0.00832382	**2.08**	Sox10,Abcb1a,Abcb1b,Spib,

					Abcg1,Abcc4
mmu00982	Drug metabolism - cytochrome P450	3.23E-04	0.00832382	**2.08**	Sox10,Mgst1,Gsto1,Ugt1a10,Spib,Cyp2d26,Ugt2b34,Gsta1
mmu01040	Biosynthesis of unsaturated fatty acids	3.61E-04	0.00832382	**2.08**	Acot2,Spib,Acot3,Acaa1b,Acot4
mmu04614	Renin-angiotensin system	8.09E-04	0.0130379	**1.88**	Sox10,Ren1,Spib,Agt
mmu00980	Metabolism of xenobiotics by cytochrome P450	8.28E-04	0.0130379	**1.88**	Sox10,Mgst1,Gsto1,Ugt1a10,Spib,Ugt2b34,Gsta1
mmu00500	Starch and sucrose metabolism	1.49E-03	0.0216274	**1.66**	Gbe1,Sox10,Ugt1a10,Spib, Ugt2b34
mmu04350	TGF-beta signaling pathway	2.31E-03	0.0312398	**1.51**	Id3,Sox10,Id2,Spib,Smad9,Smad7,Id1
mmu00533	Glycosaminoglycan biosynthesis - keratan sulfate	4.27E-03	0.0474888	**1.32**	St3gal1,Sox10,Spib
mmu00053	Ascorbate and aldarate metabolism	4.27E-03	0.0474888	**1.32**	Sox10,Ugt1a10,Ugt2b34
mmu00770	Pantothenate and CoA biosynthesis	4.27E-03	0.0474888	**1.32**	Sox10,Spib,Ppcs
mmu00280	Valine, leucine and isoleucine degradation	4.59E-03	0.0481875	**1.32**	Hmgcs2,Acaa2,Sox10,Spib, Acaa1b

NiPKO - CHOW vs. HFD – Down-regulated genes

#term	Term name	p-value	Adjusted p-value	-log(p)	Positive ids
mmu00100	Steroid biosynthesis	3.56E-08	0.000006722	**5.17**	Dhcr24, Nsdhl, Dhcr7, Tm7sf2, Sc4mol, Fdft1, Sqle,

NiPKO - CHOW vs. HFD – Up-regulated genes

#term	Term name	p-value	Adjusted p-value	-log(p)	Positive ids
mmu05340	Primary immunodeficiencies	5.96E-06	0.001126	**2.95**	Cd19,Rel,Tap2,Cd79a,Il7r,Mafb,Cd8b1
mmu04630	The Janus kinase/signal transducers and activators of transcription (JAK/STAT) pathway	7.43E-04	0.028087	**1.55**	Prlr,Ccnd2,Lep,Cebpa,Rel,Osmr,Il7r,Ccnd1,Mafb,Pik3r3,Socs2

mmu05222	Small cell lung cancer	6.13E-04	0.028087	**1.55**	Skp2,Nfkb1,Itga3,Birc2,Ccnd1,Mafb,Pik3r3,Ptgs2
mmu00260	Glycine, serine and threonine metabolism	6.04E-04	0.028087	**1.55**	Gldc,Shmt1,Aoc2,Maob,Mafb
mmu00670	One carbon pool by folate	4.31E-04	0.028087	**1.55**	Shmt1,Tyms,Mafb,Mthfs
mmu05200	Pathways in cancer	9.50E-04	0.029914	**1.52**	Fgf18,Skp2,Nfkb1,Tceb1,Egln3,Jun,Cebpa,Itga3,Fzd7,Ptch1,Nfkb2,Birc2,Brca2,Ccnd1,Mafb,Pik3r3,Ptgs2
mmu04662	B cell receptor signaling pathway	1.35E-03	0.036373	**1.44**	Nfkb1,Cd19,Jun,Cd79a,Mafb,Pik3r3,Cd22
mmu04110	Cell cycle	1.95E-03	0.041005	**1.39**	Skp2,Ccnd2,Anapc11,Wee1,Mcm4,Cdc14a,Rel,Ccnd1,Mafb
mmu04620	Toll-like receptor signaling pathway	1.87E-03	0.041005	**1.39**	Nfkb1,Jun,Cxcl10,Rel,Foxq1,Mafb,Pik3r3,Ccl3
mmu04660	T cell receptor signaling pathway	2.42E-03	0.045650	**1.34**	Nfkb1,Jun,Cebpa,Pdk1,Rasgrp1,Mafb,Pik3r3,Cd8b1
